# Genes Involved in Stress Response and Especially in Phytoalexin Biosynthesis Are Upregulated in Four *Malus* Genotypes in Response to Apple Replant Disease

**DOI:** 10.3389/fpls.2019.01724

**Published:** 2020-02-28

**Authors:** Stefanie Reim, Annmarie-Deetja Rohr, Traud Winkelmann, Stefan Weiß, Benye Liu, Ludger Beerhues, Michaela Schmitz, Magda-Viola Hanke, Henryk Flachowsky

**Affiliations:** ^1^Institute for Breeding Research on Fruit Crops, Julius Kühn-Institut, Federal Research Centre for Cultivated Plants, Dresden, Germany; ^2^Institute of Horticultural Production Systems, Woody Plant and Propagation Physiology Section, Gottfried Wilhelm Leibniz University Hannover, Hanover, Germany; ^3^Institute of Pharmaceutical Biology, Technische Universität Braunschweig, Braunschweig, Germany; ^4^Department of Natural Sciences, Hochschule Bonn-Rhein-Sieg, Rheinbach, Germany

**Keywords:** apple replant disease (ARD), gene expression, BioMark HD microfluidic system, high-throughput qRT-PCR, phytoalexins, greenhouse bio-test, soil properties, *Malus* genotypes

## Abstract

Apple replant disease (ARD) is a soil-borne disease, which is of particular importance for fruit tree nurseries and fruit growers. The disease manifests by a poor vegetative development, stunted growth, and reduced yield in terms of quantity and quality, if apple plants (usually rootstocks) are replanted several times at the same site. Genotype-specific differences in the reaction of apple plants to ARD are documented, but less is known about the genetic mechanisms behind this symptomatology. Recent transcriptome analyses resulted in a number of candidate genes possibly involved in the plant response. In the present study, the expression of 108 selected candidate genes was investigated in root and leaf tissue of four different apple genotypes grown in untreated ARD soil and ARD soil disinfected by γ-irradiation originating from two different sites in Germany. Thirty-nine out of the 108 candidate genes were differentially expressed in roots by taking a p-value of < 0.05 and a fold change of > 1.5 as cutoff. Sixteen genes were more than 4.5-fold upregulated in roots of plants grown in ARD soil. The four genes *MNL2* (putative mannosidase); *ALF5* (multi antimicrobial extrusion protein); *UGT73B4* (uridine diphosphate (UDP)-glycosyltransferase 73B4), and *ECHI* (chitin-binding) were significantly upregulated in roots. These genes seem to be related to the host plant response to ARD, although they have never been described in this context before. Six of the highly upregulated genes belong to the phytoalexin biosynthesis pathway. Their genotype-specific gene expression pattern was consistent with the phytoalexin content measured in roots. The biphenyl synthase (*BIS*) genes were found to be useful as early biomarkers for ARD, because their expression pattern correlated well with the phenotypic reaction of the *Malus* genotypes investigated.

## Introduction

Replanting apple trees at a site previously occupied by an apple plant leads to stunted shoot growth with shortened internodes, root damage, root tip necrosis, and reduction of functional root hairs (Caruso et al., 1989; Mazzola, 1998; Mazzola and Manici, 2012; Yim et al., 2013; Grunewaldt-Stöcker et al., 2019). These symptoms are referred to as apple replant disease (ARD). ARD represents a serious economic risk for tree nurseries and orchards as it leads to decreased and delayed fruit yields and reduced fruit and tree quality (Mazzola, 1998; Mazzola and Manici, 2012). At worst, a site strongly affected by ARD may become unprofitable for further apple cultivation (Geldart, 1994; Peterson and Hinman, 1994; Utkhede and Smith, 1994; Isutsa and Merwin, 2000).

Biotic agents represent the main causes of ARD as demonstrated by various disinfection experiments (Mazzola, 1998; Yim et al., 2013; Spath et al., 2015). Winkelmann et al. (2019) defined ARD as “a harmfully disturbed physiological and morphological reaction of apple plants to soils that faced alterations in their (micro)biome due to previous apple cultures.” There is substantial evidence that the changes in the soil biota trace back to root exudates and material from decomposing apple roots (Börner, 1959; Wittenmayer and Szabó, 2000; Hofmann et al., 2009; Winkelmann et al., 2019). Up to now, no practicable counteraction against ARD is available. The most employed countermeasures, crop rotation and soil disinfection, are unfeasible due to either environmental hazards or high costs (Winkelmann et al., 2019). In order to develop novel strategies against ARD, both the reactions of the apple plant and the etiology of the disease should be understood in more detail (Weiß et al., 2017b; Winkelmann et al., 2019).

Recent transcriptomic analyses revealed the induction of genes associated with biotic stress response in roots of apple plants grown in ARD soil (Weiß et al., 2017a; Weiß et al., 2017b). This corresponds well with the findings, that parasitic fungi and oomycetes of the genera *Cylindrocarpon* (Tewoldemedhin et al., 2011b; Mazzola and Manici, 2012; Franke-Whittle et al., 2015; Manici et al., 2015), *Phytophthora* (Tewoldemedhin et al., 2011a; Mazzola and Manici, 2012), *Pythium* (Tewoldemedhin et al., 2011a; Mazzola and Manici, 2012; Manici et al., 2013), and *Rhizoctonia* (Tewoldemedhin et al., 2011a; Mazzola and Manici, 2012; Manici et al., 2013) are enriched in ARD soil in comparison to healthy or disinfected soil. In particular, genes of the biphenyl biosynthetic pathway were rapidly activated in the roots of apple plants grown in ARD soil (Zhu et al., 2014; Zhu et al., 2016; Weiß et al., 2017a; Weiß et al., 2017b). Phytoalexins like biphenyls and dibenzofurans are known to act in an induced defense mechanism against biotic stressors, such as fungi and bacteria (Ahuja et al., 2012; Chizzali et al., 2012b; Chizzali and Beerhues, 2012; Chizzali et al., 2013). Interestingly, the activation of phytoalexin biosynthesis genes was also found when the plants were inoculated with *Pythium ultimum*, one component of the ARD complex (Shin et al., 2016b; Zhu et al., 2016). Along with the elevated gene expression, individual phytoalexin compounds were found in higher abundances in roots growing in ARD soils (Weiß et al., 2017b).

Additional phenolic compounds play a role in the ARD response, which were shown to accumulate in response to infected soil and may indicate the occurrence of oxidative stress (Henfrey et al., 2015). Especially the dihydrochalcones phloridzin and phloretin have been found highly abundant in apple root exudates and root debris (Hofmann et al., 2009; Emmett et al., 2014; Nicola et al., 2016; Leisso et al., 2018). They act against pathogens and as scavengers of reactive oxygen species (Börner, 1959; Emmett et al., 2014; Henfrey et al., 2015). An upregulation of flavonol metabolism genes was also found in apple roots under replant conditions (Weiß et al., 2017a; Weiß et al., 2017b) and upon infection with *P. ultimum* (Shin et al., 2014; Zhu et al., 2014; Shin et al., 2016b; Zhu et al., 2019).

Further genes upregulated under ARD conditions are involved in auxin, ethylene, jasmonate, and cytokinin biosyntheses and signaling (Shin et al., 2014; Shin et al., 2016b; Weiß et al., 2017a; Zhu et al., 2019). Salicylic acid, ethylene, and jasmonic acid are important signaling compounds in the biotic stress defense response (Glazebrook, 2005; Broekaert et al., 2006). Moreover, ethylene can induce the biosynthesis of phytoalexins derived from the phenylpropanoid pathway (Kamo et al., 2000; Chung et al., 2001; Ishigaki et al., 2004). Biotic stress signaling involves the activation of signal transduction pathways and the activation of a number of transcription factors. As plant shoot and root growth are strongly altered by ARD, changes in auxin, cytokinin, abscisic acid, and gibberellin homeostasis and signaling are expected to occur.

In this study, we compared the expression of 108 candidate genes (CGs) that were supposed to be involved in the reaction of apple to ARD soil. The majority of these CGs were selected from the transcriptomic data available from Weiß et al. (2017a 2017b)) and Weiß and Winkelmann (2017). These CGs were shown to be differentially expressed in roots and leaves of the ARD-sensitive apple rootstock M26 grown in untreated ARD soil and disinfected ARD soil. Further CGs were chosen based on the literature with a focus on the following functional categories: flavonoid biosynthesis, oxidation–reduction processes, jasmonic acid–mediated signaling and responses to wounding, defense, and auxin metabolism (Dal Cin et al., 2009; Milcevicova et al., 2010; Devoghalaere et al., 2012; Dugé De Bernonville et al., 2012; Shin et al., 2014; Qian et al., 2016; Shin et al., 2016a). CG expression was compared between four apple genotypes with different genetic background and different susceptibility/tolerance towards ARD. The apple genotypes were grown in a bio-test using ARD soil from two different ARD sites.

The objectives of the present study were: (I) to evaluate the expression of 108 CGs in response to ARD in roots and leaves of plants tested in a greenhouse bio-test using a high-throughput microfluidic approach, (II) to determine the influence of the *Malus* genotype on the quantitative expression of the CGs, and (III) to correlate the gene expression data to both the ARD severity measured in the bio-test employing two different ARD soils and the phytoalexin contents detected in roots. The results provide new insights into genotypic differences in the complex reaction to ARD and give new hints to mechanisms contributing to ARD sensitivity or tolerance.

## Material And Methods

### Soil Origin and γ-Irradiation

ARD soil from the two sites Heidgraben (53°41'57.5"N, 9°40'59.6"E) and Meckenheim (50°37'8.5"N, 6°59'25.4"E) was sampled at a depth of 0–20 cm. The soil from Heidgraben is an entic podzol, and that from Meckenheim was classified as a haplic luvisol developed from loess (Mahnkopp et al., 2018). The detailed soil properties are described in **Table 1**. On the sampled Heidgraben plots, ARD had been induced by four times replanting of *Malus domestica* Borkh. cv. ‘Bittenfelder’ as described in detail by Mahnkopp et al. (2018). The Meckenheim site has been in use for apple variety tests grafted on the rootstock M9 since 2006. Replanting took place in the years 2010 and 2017 (G. Baab and L. von Schoenebeck, personal communication).

**Table 1:** Major properties of soils from the two apple replant disease (ARD) sites Heidgraben and Meckenheim at 0–20 cm depth.

Site	Particle size distribution			SOC [g kg^−1^]	N_total_ [g kg^−1^]	pH (CaCl_2_)	CaCO_3_ [g kg^−1^]
	Sand [%]	Silt [%]	Clay [%]				
Heidgraben	92.9	2.8	3.1	25.4	1.54	5.3	<0.1
Meckenheim	6.9	72.0	21.1	12.3	1.5	6.7	<0.1

Particle size distribution, total carbon and nitrogen (N_total_) are displayed. Soil organic carbon (SOC) represents total carbon due to the absence of carbonate (CaCO_3_).

Both soils were sieved through an 8 mm mesh. Half of each soil volume was filled into autoclavable bags in portions of 10–15 L. The soil was γ-irradiated with a minimum dose of 10 kGy (recorded dosages: minimum 10.87 kGy, maximum 31.96 kGy, BGS Beta-Gamma-Service, Wiehl, Germany) by which most fungi, bacteria, and invertebrates are killed (McNamara et al., 2003). Hereafter, the untreated ARD soil will be denoted as ARD soil and the ARD soil disinfected by γ-irradiation as γARD soil.

The effect of the γ-irradiation was confirmed by plating diluted soil solutions on growth media selective for bacterial or fungal growth (Balbín-Suárez et al., personal communication). Bacterial colony-forming units (CFUs) were counted after 2 days and fungal CFUs after 7 days. Briefly, 9 mL of 0.85% NaCl solution (saline) were added to 1 g of soil under sterile conditions and vortexed for 2 min. After settling of the soil particles, serial dilutions (factor 10) of the supernatant were made by mixing 100 µL soil solution with 900 µL saline. For each of the four soil variants, two samples were taken for plating. For the γ-irradiated soil samples, 100 µL of the 1:10 and 1:100 dilution were plated; for the untreated soil variants, dilutions 1:100, 1:1,000, and 1:10,000 were plated. Each plating was carried out in triplicates. The culture media used were Reasoner's 2A agar (R2A agar, Carl Roth, Karlsruhe, Germany) supplemented with 100 mg L^−1^ cycloheximide for bacteria and Potato Dextrose Agar (PDA, Merck, Darmstadt, Germany) supplemented with 100 mg L^−1^ penicillin, 10 mg L^−1^ tetracycline, and 50 mg L^−1^ streptomycin for fungi. The plating was carried out twice, before and after storage of the γ-irradiated and untreated soils, to evaluate an effect of the storage (**Table S3**).

### Plant Material and Experimental Setup

Plants of the apple genotypes M26, M9, B63, and the *Malus × robusta* accession MAL0595 were used. B63 is an offspring of the cross (*M. purpurea* ‘Eleyi’ *× M. sieboldii*) *×* M9 and was derived from a breeding program for resistance to apple proliferation disease (W. Jarausch, personal communication). The accession MAL0595 was derived from the *Malus* gene bank collection of the Julius Kühn-Institut (Dresden-Pillnitz, Germany). All genotypes were propagated *in vitro* via axillary shoots on a modified MS medium (Murashige and Skoog, 1962) containing 3% sucrose, 0.5 µM indole-3-butyric acid (IBA), and 4.4 µM 6-benzylaminopurine (BAP). For M9, 2 mL L^−1^ Plant Preservative Mixture (PPM, Plant Cell Technology, Washington DC, USA) was added to the culture medium in order to control growth of endophytic bacteria. MAL0595 subculture was carried out once with MS medium containing 3% sucrose, 0.5 µM IBA, and 4.54 µM Thidiazuron (TDZ) to increase the number of shoots obtained. All *in vitro* cultures were incubated at 24°C with a 16 h photoperiod provided by Philips MASTER TL-D 58W/865 fluorescence tubes at a PPFD (Photosynthetic Photon Flux Density) of 35–40 µmol m^−2^ s^−1^.

*In vitro* rooting was induced by transferring the 5-week-old shoots to ½ MS medium supplemented with 2% sucrose and 4.92 µM IBA (Weiß et al., 2017a). The rooting percentages determined 2 weeks after transfer to rooting medium were 95.8% for M26 (n = 168), 8.9% for M9 (n = 168), 64.8% for B63 (n = 168), and 31.4% for MAL0595 (n = 242) respectively. All plants were transferred to peat substrate (Steckmedium, Klasmann-Deilmann GmbH, Geeste, Germany). For acclimatization, the shoots were cultivated under covers to ensure high humidity. During acclimatization, the plants were adapted to greenhouse conditions by gradually reducing the air humidity. After about 4 weeks, the plants were introduced to the bio-test. ARD and γARD soils from Heidgraben and Meckenheim were supplemented with 2 g L^−1^ of the slow-release fertilizer Osmocote Exact 3-4 M (16-9-12+2MgO+trace elements, https://icl-sf.com) and filled into fifteen 0.4 L pots for gene expression samples and ten 1 L pots per soil variant and genotype for growth parameters. The M9 rootstock was tested with only 12 plants due to poor rooting and acclimatization, 6 in Heidgraben ARD soil and 6 in γARD soil in 0.4 L pots for gene expression analysis.

The greenhouse experiment took place from August 9, 2017, to September 7, 2017, at the campus Herrenhausen (Gottfried Wilhelm Leibniz University Hannover, Hanover, Germany). All 312 plants were randomly arranged and cultivated at 22.4 ± 2.8°C and a relative air humidity of 68.2 ± 8.2%. Additional light was provided whenever solar irradiation fell below 25 klx to provide 16 h of daylight. Plant protection against thrips was carried out according to horticultural practice. Shoot length was measured on a weekly basis.

After 7 days, all plants for gene expression analysis were harvested and carefully removed from the soil. Whole root systems were washed gently in tap water and dried with paper towels, and the three youngest fully developed leaves were sampled. Root and leaf samples were transferred to 2 mL reagent tubes, immediately frozen in liquid nitrogen, and stored at −80°C until RNA isolation.

Ten plants per variant representing 10 biological replicates (except of M9) were harvested after 4 weeks for determining growth parameters. Single plants died off (resulting in only nine biological replicates) from the following variants (**Table S1**): MAL0595 in Heidgraben ARD, B63 in Meckenheim ARD, and MAL0509 in Meckenheim γARD. Plant quality was assessed visually by inspection of root color and habitus. Plant growth was determined by measuring shoot length as well as shoot and root fresh masses. Roots of four to five plants per variant were lyophilized for 3 days and used for dry mass evaluation and phytoalexin analysis after freeze-drying for 3 days.

### RNA Isolation and First Strand cDNA Synthesis

From 15 plants of each of the genotypes M26, B63, and MAL0595, five pools containing 3 single plants each, i.e. five biological replicates, were established for each of the four soil variants. For M9, only 12 plants were available. These plants were grown in Heidgraben soil, six in γARD soil and six in ARD soil, respectively. For M9, two pooled samples containing three plants each were created for each of the two soil variants. Selection of the plants for each pool was carried out with regard to shoot length to achieve a similar mean shoot length among the pools.

The pooled samples were homogenized in a Mixer Mill at 27 Hz for 1 min (Mixer Mill MM400, Retsch, Haan, Germany) cooled with liquid nitrogen. Total RNA was extracted from 100 mg of frozen ground plant material with RP lysis buffer using the InviTrap Spin Plant RNA Mini Kit (Stratec, Birkenfeld, Germany) according to the manufacturer's instructions. Genomic DNA was removed with DNase I (Thermo Scientific, Waltham, MA, USA) following the manufacturer's instructions. RNA concentration and quality was determined spectrophotometrically (NanoDrop 2000c, Peqlab, Erlangen, Germany). The integrity was checked on a 1% agarose gel. The isolated RNA was stored at −80°C until first strand cDNA synthesis using the RevertAid First Strand cDNA Synthesis Kit (Thermo Scientific, Waltham, MA, USA) together with oligo dT primers and 1 µg RNA as template. The resulting cDNA was diluted 10-fold in nuclease-free water and stored at −20°C until use. The success of cDNA synthesis and the exclusion of genomic DNA contaminations was verified in a standard PCR with the primer pair EF1-for/-rev (EF1-for ATTGTGGTCATTGGYCAYGT; EF1-rev CCAATCTTGTAVACATCCTG) using 1 µl of the diluted cDNA as well as 1 µl of the RNA preparation (Boudichevskaia et al. (2009). PCR products resulting from genomic DNA and cDNA differ in fragment size (905bp/707bp), whereas no product should be generated using RNA.

### Primer Selection and RT-qPCR Validation

The CG primer set was compiled on the basis of genes differentially expressed in root and leaf material of *Malus* rootstock M26 grown in ARD soil compared to γARD soil (Weiß et al. 2017a; Weiß et al. 2017b; Weiß and Winkelmann, 2017). Additionally, known pathogen and stress-related genes focusing on plant hormone signaling of *Malus* and *Arabidopsis thaliana* described in the literature were selected (Dal Cin et al., 2009; Milcevicova et al., 2010; Devoghalaere et al., 2012; Dugé De Bernonville et al., 2012; Shin et al., 2014; Qian et al., 2016; Shin et al., 2016a; Weiß et al., 2017a; Weiß et al., 2017b). A full list of all primers is provided in **Table S2**. All primers were validated *in silico* using the software program FastPCR v6.6 (PrimerDigital Ltd, Helsinki, Finland) (Kalendar et al., 2017) by calculating theoretical PCR results using the *Malus* × *domestica.*v1.0.consensus_CDS database obtained from http://www.rosaceae.org. The program predicted possible PCR products with a length of 50–3,000 bp, with one mismatch allowed at the 3'-end.

New primers were designed using the Primer3 web tool with the following parameters: primer length 18–24 bp, amplification product 100–200 bp, T_M_ = 59–61°C, CG content 40–60%. The specificity of the new primers was also tested *in silico* as described. Primer sequences with proven specificity to the target gene sequence were checked for sufficient amplification efficiency with RT-qPCR. The *Elongation factor 1-α* [MDP0000304140], *Elongation factor 1β-like* [MDP0000903484], *Tubulin beta chain* [MDP00009551799], *Ubiquitin-conjugating enzyme E2 10-like* [MDP0000140755], and *Actin-7* [MDP0000774288] were used as reference genes according to Weiß et al. (2017a). Each primer combination (75 nM each primer) was analyzed with three technical replicates using the Maxima SYBR Green master mix (Thermo Fisher Scientific, Schwerte, Germany). All primers were tested at an annealing temperature of 60°C and cDNA of the apple rootstocks M9, M26, CG41 and the wild apple genotype *Malus* × *robusta* 5 (accession no. MAL0991) grown in untreated ARD soil, since no cDNA of B63 and MAL0595 was available at this time. RT-qPCR was performed on an iCycler iQ Real Time PCR Detection System (Bio-Rad) with an initial denaturation of 3 min at 94°C followed by 40 cycles of 1 min at 94°C, 1 min at 60°C, and 1 min at 72°C. The PCR products were analyzed by melt-curve analysis of 55°C to 80°C with an increment of 0.5°C for 10 s each step. Data were recorded with the software package Genex (Bio-Rad, München, Germany). PCR efficiencies were calculated using the software program LinRegPCR (Ramakers et al., 2003; Ruijter et al., 2009). The PCR efficiencies presented in **Table S2** are mean values of all samples per primer combination, where expected amplicons (based on melting temperature) were detectable. Primer pairs producing more than one distinct peak in the melt-curve analysis were assigned as not specific. These primers were rejected from further RT-qPCR analysis.

To test the specificity of the primers used to amplify the genes *B4Ha* and *B4Hb* (**Table S2**), an amplicon deep sequencing was conducted. The sequence analysis proved the *B4Hb* primers to be highly specific. The sequencing results also showed that the *B4Ha* amplicon is present in both *B4Ha* and *B4Hb*. This means that the primers for B4Ha are not gene-specific. This limited specificity should be considered for the interpretation of the respective data.

### Expression Analysis Using Quantitative PCR

RT-qPCR was performed using the BioMark HD high-throughput system (Fluidigm, South San Francisco, California, USA) by analyzing 128 individual samples, consisting of 64 root and leaf samples respectively, with 116 primer pairs (including 5 primer pairs for reference genes) using six Dynamic Array™ integrated fluidic circuits (96.96 IFCs, Fluidigm, South San Francisco, California, USA). The sample design included five biological replicates for each genotype (B63, M26 and MAL0595), soil treatment, and soil origin. For M9, only samples of the Heidgraben soil were analyzed with only two replicates of each soil treatment (ARD/γARD). The entire analysis included two technical repetitions for each biological replicate. Default space on these IFCs allowed the analysis of 96 samples with 96 primers in one run.

For specific target amplification, 1.25 µL cDNA was pre-amplified in a mixture with 0.5 µL of pooled primers (final concentration, 500 nM), 2.5 µL of 2× PreAmp Master Mix (Applied Biosystems, Carlsbad, CA, USA), and 0.75 µL of water. The cycling program was as follows: 95°C for 10 min, followed by 14 cycles of 95°C for 15 s and 60°C for 4 min. Afterwards, the PCR reactions were purified with exonuclease (20 U µL^−1^) and diluted 1:5 with Teknova-DNA suspension buffer (VWR, Darmstadt, Germany). The qPCR was performed in 96.96 Dynamic Array™ IFCs (Fluidigm, South San Francisco, CA, USA) following the manufacturer's instructions. Each assay inlet contained 5 µL of an assay mix consisting of 0.5 μM primer mix, 2.5 µL assay loading reagent (Fluidigm), and 2.25 µL 1× TE buffer assay reagent. The Fluidigm sample premix contained 2.25 µL of the pre-amplified sample, 2.5 µL of 2× SsoFast EvaGreen supermix with low ROX (Bio-Rad, München, Germany), and 0.25 µL of 20× Binding Dye Sample Loading Reagent (Fluidigm). The cycling program was: 1 min at 95°C, followed by 30 cycles of 96°C for 5 s and 20 s at 60°C plus melting curve analysis.

### Extraction and Analysis of Phytoalexins

At the final evaluation of the experiment (4 weeks after potting), the root systems of the genotypes M26, B63 and MAL0595 were combined to obtain two pools (i.e. two biological replicates composed of roots of four to five plants) per soil variant. The roots were lyophilized for 3 days (alpha 1-2 LDplus, Christ, Osterode, Germany). The dry roots were homogenized in a mixer mill (Mixer Mill MM400, Retsch, Haan, Germany) with steel beads. Before phytoalexin extraction, 4-hydroxybiphenyl (50 µg) was added to each sample (around 100 mg DW each) as internal standard for quantification in gas chromatography–mass spectrometry (GC-MS) measurement. The samples were extracted with 1 mL methanol by shaking in a Vortex Genie 2 (Scientific Industries, Bohemia, NY, USA) for 20 min. The extracts were centrifuged at room temperature at 13,000 rpm for 10 min. An aliquot of the supernatant (200 µL) was transferred to a new 1.5 mL Eppendorf tube and dried under a constant air stream. The residue was re-suspended in 200 µL ethyl acetate and centrifuged at 13,000 rpm for 10 min. The resulting clear supernatant was transferred to a GC-MS vial with a glass inlet. After removal of the ethyl acetate by air stream, 50 µL N-trimethylsilyl-N-methyl trifluoroacetamide (MSTFA) was added to the inlets for derivatization at 60°C for 30 min. The samples were then measured by GC-MS, as described previously (Hüttner et al., 2010).

### Data Analysis and Statistical Evaluation

A mean PCR efficiency (quality score) was calculated using the Fluidigm Real-Time PCR Analysis Software v4.3.1 (Fluidigm, South San Francisco, CA, USA). Therefore, each individual amplification curve was compared to an ideal exponential curve. The closer the amplification curve is to the ideal, the quality score approaches 1. The further the curve is from ideal, the quality score approaches 0. Only quality score values above 0.65 (an arbitrary threshold set by Fluidigm) passed the quality check. Curves that fail the quality threshold were excluded from further calculations. Considering the quality threshold and the quantification cycle (Cq), separate ΔCq values for sample and control were calculated. This was done on basis of the following formulas:

Sample ΔCq=ΔCq Candidate gene (ARD soil)−ΔCq reference gene (ARD soil)

Control ΔCq=ΔCq Candidate gene (γARD soil)−ΔCq reference gene (γARD soil)

The reference genes were validated according to their stability using NormFinder (Andersen et al., 2004). All reference genes with stability values below 0.25 were included in the ΔCq value calculation, so that depending on the IFC, three to five reference genes were considered in the control ΔCq calculation. The ΔΔCq value was calculated by subtracting the control ΔCq value from the sample ΔCq value, which resulted in the relative gene expression (fold change, 2^−ΔΔCq^) (Livak and Schmittgen, 2001). Throughout this paper, gene expression is presented as relative expression level in ARD soil compared to the expression in γARD soil, which was set to be one.

The test for normal distribution was carried out with the Shapiro–Wilk test using SAS version 9.4 (SAS, NC, USA). The effect on gene expression of different soil treatments (ARD soil and γARD soil) was tested with the analysis of variance (ANOVA) also using SAS version 9.4. Furthermore, the effect of genotype and soil origin (type) on gene expression was tested using the ANOVA procedure MIXED in SAS version 9.4. The STRING database (Szklarczyk et al., 2017) was used to predict the interaction of the detected differentially expressed genes (DEGs).

Data on shoot length, fresh and dry masses, and phytoalexin content were evaluated using R version 3.5.1 (R Development Core Team, 2011) in R Studio version 1.1.45. The data were checked for a Gaussian distribution and log transformed, if necessary. A linear model was fitted for each parameter, and an ANOVA was calculated. Multiple comparisons of means (Tukey test) were carried out using the R package “multcomp” version 1.4-8 (Hothorn et al., 2008).

Using the software program SAS version 9.4. Pearson's correlation was analyzed between the phenotypic data (shoot length and fresh biomass) and the fold change values of selected phytoalexins as well as CGs.

## Results

### Phenotyping of the Genotypes After 4 Weeks

Plating of the soil solution proved the success of the soil disinfection with the significant reduction in bacterial and fungal colony-forming units (CFUs) (**Table S3**). Plant growth of the genotypes M26, B63, and MAL0595 was negatively affected by ARD. After 4 weeks of cultivation in ARD soil, shoots were smaller with lower biomasses for B63 and M26, but not for MAL0595 (**Tables 2** and **S2**). The reduction in shoot length was stronger in Meckenheim soil than in Heidgraben soil (**Table 2**).

**Table 2:** Shoot length and fresh mass of shoot and root of M26, B63, and MAL0595 4 weeks after transplanting to γARD soil and ARD soil from Heidgraben and Meckenheim.

Genotype	Shoot length	Heidgraben				Meckenheim			
		γARD soil	ARD soil	% red		γARD soil	ARD soil	% red	
B63	[cm]	4.1 b	2.8 a	**−32.4**	**	7.4 c	3.0 a	**−60.2**	***
M26		3.5 b	2.5 a	**−30.0**	*	5.9 c	3.6 b	**−39.8**	***
MAL0595		5.1 a	4.5 a	**−11.6**	n.s.	6.2 a	4.4 a	**−28.0**	n.s.
Genotype	Fresh biomass **shoot**	**Heidgraben**				**Meckenheim**			
		**γARD soil**	**ARD soil**	**% red**		**γARD soil**	**ARD soil**	**% red**	
B63	[g]	0.95 b	0.54 a	**−42.8**	**	1.40 c	0.71 ab	**−49.3**	***
M26		1.00 b	0.60 a	**−39.8**	*	1.57 c	0.81 ab	**−48.1**	***
MAL0595		0.81 ab	0.48 b	**−41.2**	n.s.	1.47 a	0.88 ab	**−39.9**	n.s.
Genotype	Fresh biomass **root**	**Heidgraben**				**Meckenheim**			
		**γARD soil**	**ARD soil**	**% red**		**γARD soil**	**ARD soil**	**% red**	
B63	[g]	0.42 b	0.24 a	**−41.3**	*	0.35 ab	0.21 a	**−37.8**	n.s.
M26		0.27 c	0.16 ab	**−40.2**	**	0.23 bc	0.12 a	**−48.2**	***
MAL0595		0.36 a	0.20 a	**−42.7**	n.s.	0.31 a	0.36 a	**19.5**	n.s.

Mean values with the same letter within one row did not differ significantly (Tukey test, *p* = 0.05, n = 9–10). Reduction (%, red) compared between γARD soil and ARD soil from one site is given in bold. Asterisks indicate a significant reduction regarding the Tukey test [*p* < 0.05 (*), *p* < 0.01 (**), and *p* < 0.001 (***); n.s. = not significant].

**Figure 1:**
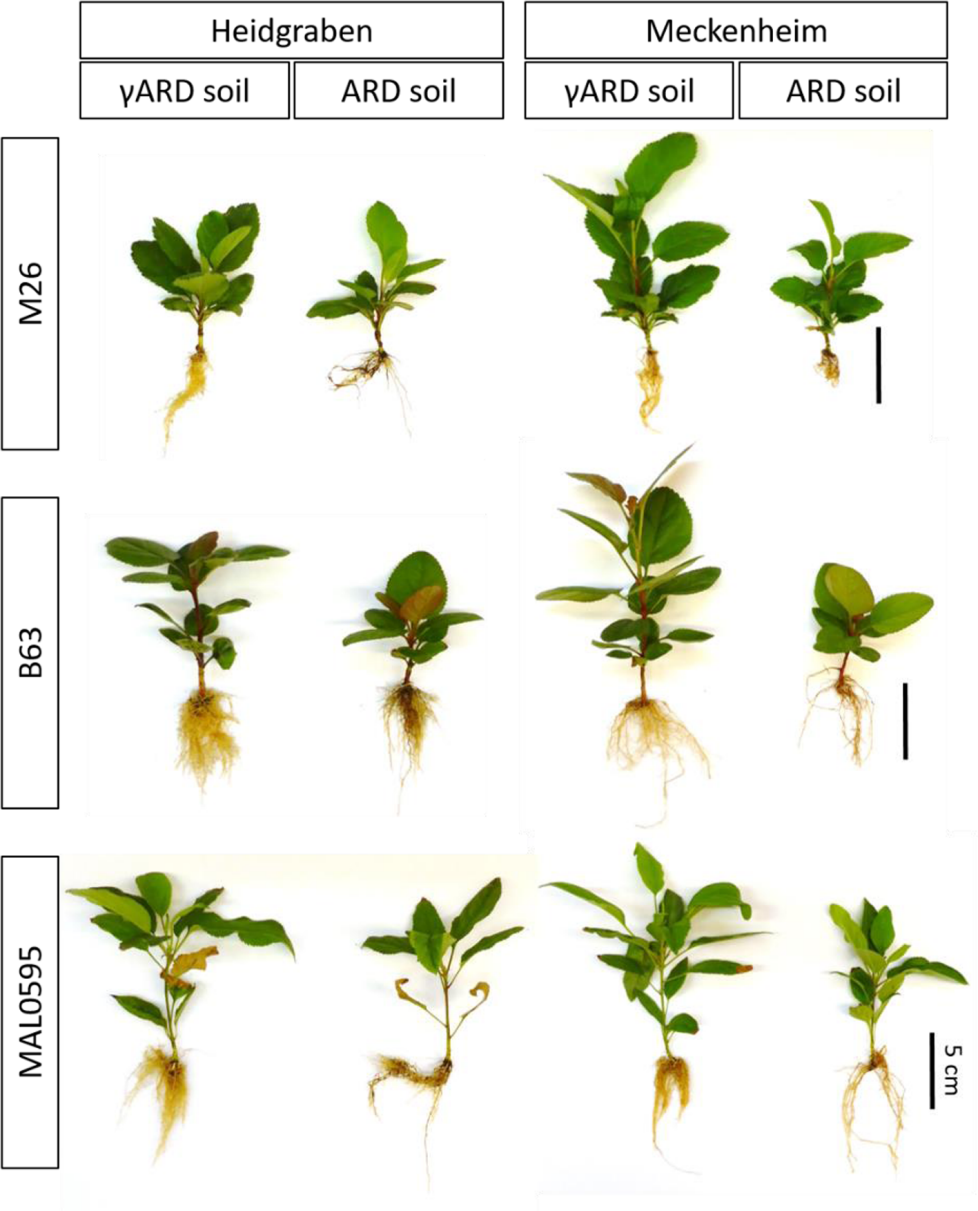
Apple plants of M26, B63 and MAL0595 4 weeks after planting to γ-irradiated apple replant disease (γARD) soil and ARD soil from the sites Heidgraben and Meckenheim.

As depicted in **Figure 1**, the roots of all three genotypes showed a darker coloration when grown in ARD soil from both sites. In addition, less fine roots were visible in the ARD variants. The rootstock M9 was not included in this final evaluation, as only a few plants were available.

Shoot and root fresh biomass of B63 and M26, were significantly reduced on ARD soil from both sites. For MAL0595, the change in fresh biomass was not significant. In Meckenheim soil, M26, and B63 showed a stronger reduction in shoot and root biomass compared to Heidgraben soil (**Table 2**). Generally, a higher shoot fresh biomass was achieved by plants grown in Meckenheim soil as seen by the control plants grown in γARD soil from this site. A similar pattern was found for the fresh root biomasses. A significant reduction was observed for M26, with a stronger effect in Meckenheim soil. MAL0595 root biomass did not differ significantly between the treatments, and in Meckenheim soil, the reduction was approximately halved compared to B63 and M26, (**Table 2**). For B63, root biomass was not significantly reduced when grown in Meckenheim ARD soil.

Although MAL0595 showed comparable reduction in shoot and root biomass with B63 and M26 (except root biomass in Meckenheim soil), this reduction was statistically not significant. One explanation for that is the high variation between individual plants from the same genotype.

### Establishment of Gene-Specific Primers

Primer pairs for 122 genes (117 CGs and 5 reference genes) were tested *in silico* against the *Malus* × *domestica*.v1.0.consensus_CDS database (**Table S2**). Thirty-nine combinations showed unspecific amplification. Redesign of new primer combinations was successful for 33 out of these genes. For six genes, no gene-specific primers were found. These genes were excluded from subsequent analyses (**Table S4**). In total, 111 primer pairs (CGs only) were tested for their amplification efficiency by RT-qPCR (**Table S2**). The PCR efficiencies varied between 1.77 and 2.10 (a value of 2 is equal to an amplification efficiency of 100%). After melt-curve analysis, 108 primer combinations were confirmed as highly specific, whereas the specificity of three combinations (*IPT*, *Mal d1.06*, and *FGT*) was insufficient. For four primer combinations (*NTL9*, *PDF2.2*, *ABCB11b*, and *Bax_inh*) the melting temperature varied slightly. These ranges were detectable between individual samples of the same tissue of the same genotype. On this account, the amplicons were most likely derived from the same gene and not from different orthologous sequences.

### Genes Differentially Expressed in Response to ARD

Gene expression of 108 CGs was analyzed in leaf and root tissue of B63, M26, M9, and MAL0595. For this, plant material was collected after 7 days of cultivation in four different soil variants. The relative gene expression (ARD soil vs. γARD soil) ranged from 0.5-fold to 31.9-fold (**Table S5**). Fourteen CGs were slightly downregulated in plants grown in ARD soil compared to those grown in γARD soil (10 genes in roots, 3 in leaves, and 1 in leaves and roots). Out of the 108 CGs, 42 DEGs were identified by taking a p-value of < 0.05 and a fold change of greater than 1.5 as cutoff (**Table S6**). Thirty-nine genes were differentially expressed in roots. Thirty-one of them were only differentially expressed in roots, whereas eight genes were upregulated in both tissues. The remaining four genes were differentially expressed in leaves only.

### Highly Regulated CGs with a Significant Fold Change > 4.5

**Figure 2:**
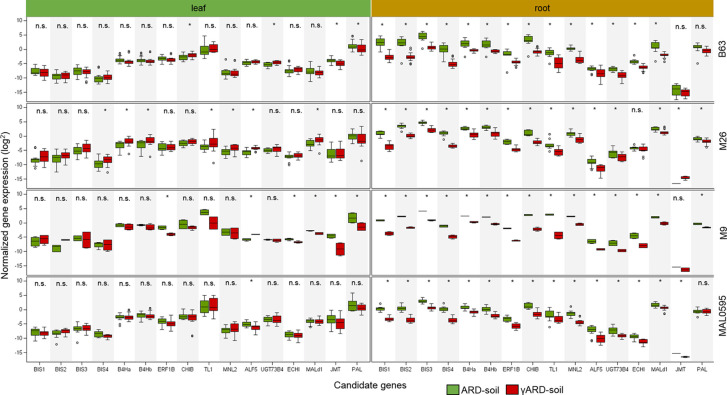
Normalized gene expression values in leaf or root tissue of 16 highly upregulated candidate genes (CGs) in four different apple genotypes (B63, M26, M9 and MAL0595) grown in ARD and γARD soil, respectively. The normalized gene expression values are average values for the both soil origins (Meckenheim and Heidgraben). Non-significant values are indicated as n.s.; significant values (*p* < 0.05) are indicated with *. *BIS1*, biphenyl synthase 1; *BIS2*, biphenyl synthase 2, *BIS3*, biphenyl synthase 3; *BIS4*, biphenyl synthase 4; *B4Ha*, biphenyl 4-hydroxylase isoform a; *B4Hb*, biphenyl 4-hydroxylase isoform b; *ERF1b*, ethylene-responsive transcription factor 1B-like; *CHIB,* endochitinase EP3-like; *TL1*, thaumatin-like protein 1a; *MNL2*, putative mannosidase; *ALF5*, multi antimicrobial extrusion protein; *UGT73B4*, uridine diphosphate (UDP)-glycosyltransferase 73B4; *ECHI*, chitin-binding type 1; *Mal d1*, major allergen Mal d1-like; *JMT*, jasmonate O-methyltransferase-like; *PAL*, phenylalanine ammonia-lyase.

**Figure 3:**
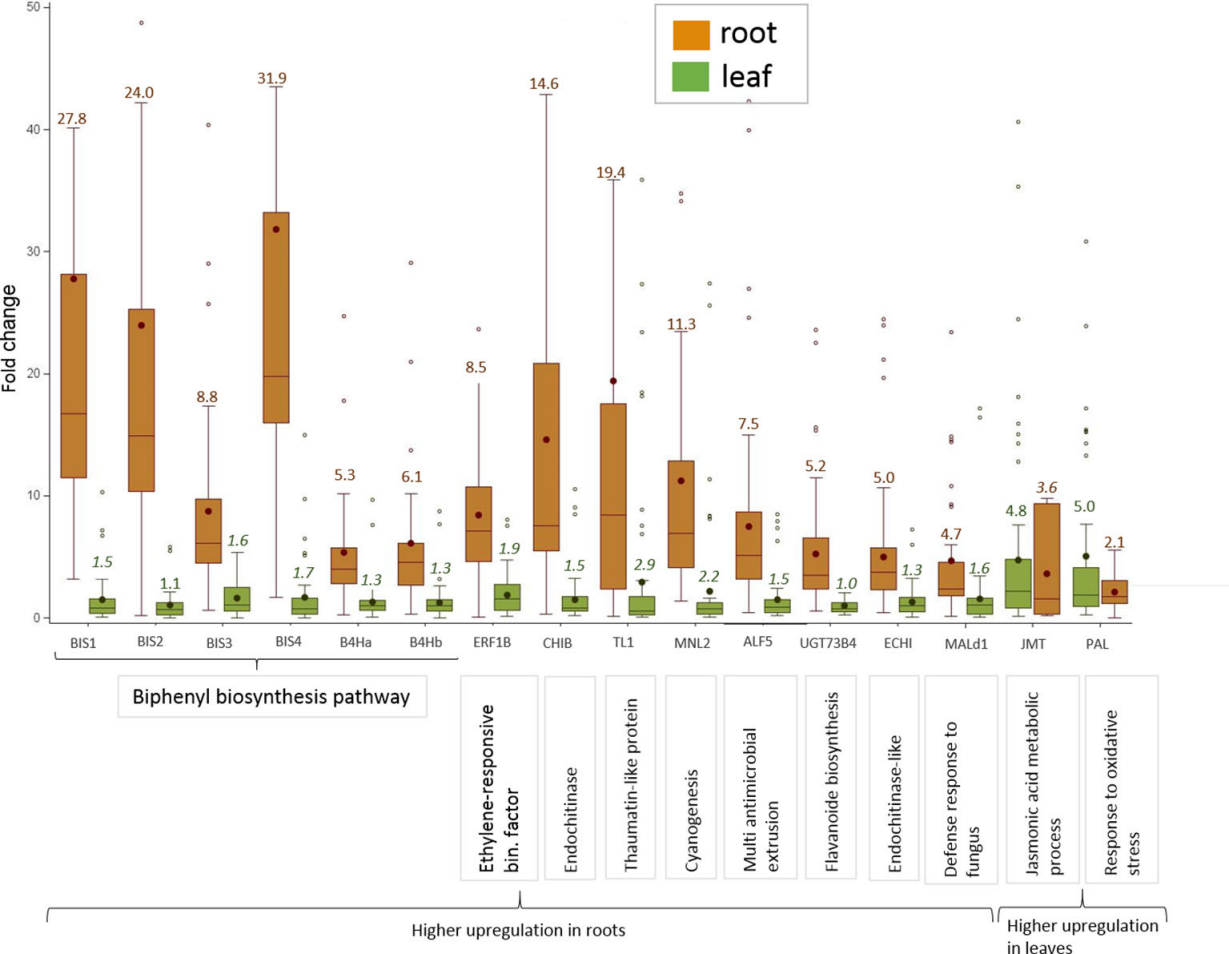
Highly upregulated CGs in apple grown in ARD soils with an average ΔΔCq-fold change value > 4.5 in either root or leaf tissue after cultivation in ARD soil and their assignment to molecular function. The fold change values (ARD soils/γARD soils) are average values for the four genotypes, including five (M26, B63 and MAL0595) or two replicates (“M9”), respectively and the two soils (Meckenheim and Heidgraben). Non-significant fold change values are indicated in italics. The whiskers were not drawn to the minimum or maximum values, if they were longer than 1.5 times the interquartile range (IQR). Data points outside of this range of 1.5 × IQR were indicated as outliers (dots).

Sixteen CGs were highly, i.e. more than 4.5-fold, upregulated in roots of plants growing in ARD soil compared to those growing in γARD soils. Fourteen of them were significantly upregulated in root tissue in all four apple genotypes (**Figure 2**). Six of them (*BIS1, BIS2, BIS3, BIS4, B4Ha,* and *B4Hb*) belong to the phytoalexin biosynthetic pathway, whereas one gene (*ERF1B*) is a transcription factor binding to a pathogenesis-related element and an additional gene belongs to the endochitinase family (*CHIB*). The six remaining genes are associated with six gene families of different biological functions (**Figure 3**). The highest upregulation of gene expression in roots grown in ARD soils was detected for the phytoalexin biosynthesis genes. The average fold changes were 31.9 for *BIS4*, 27.8 for *BIS1*, and 24.0 for *BIS2*. In contrast, *BIS3* was only 8.8 times more highly expressed in ARD soil than in γARD soil, but this gene showed the overall highest expression level (**Figure 2** and **Table S5**). The two further genes of this pathway, *B4Ha* and *B4Hb*, were upregulated after cultivation in ARD soil with fold changes of 5.3 and 6.1, respectively.

Three genes upregulated in roots of all four genotypes after cultivation in ARD soils seem to be involved in regulating the molecular response to pathogen attack and/or plant defense. The chitinase B gene *CHIB* showed a significant fold change value of 14.6 in all root samples of plants grown in ARD soils. The gene *TL1* encoding the thaumatin-like protein was also upregulated in root samples with a significant fold change value of 19.4. The putative mannosidase gene *MNL2* being involved in cyanogenesis and defense response was upregulated with an average fold change of 11.3. For the ethylene-responsive transcription factor 1B-like (*ERF1B*), a fold change value of 8.5 was detected.

Within the multi antimicrobial extrusion protein family, an average fold change of 7.5 was detected in root samples for the gene *ALF5*. A significant upregulation (fold change 5.0, *p* < 0.05) was also detected for the chitin-binding type 1 gene (*ECHI*), which belongs to the endochitinase-like superfamily. Within the multigene family of plant glycosyltransferases, the uridine diphosphate (UDP)-glycosyltransferase (UGT) 73B4 encoding gene *UGT73B4* showed a 5.2-times higher expression in root tissue in ARD soils. The gene *Mal d1* encoding the major allergen Mal d1 showed an increased expression in root tissue with a significant fold change of 4.7.

The genes for jasmonate O-methyltransferase-like (*JMT*) and phenylalanine ammonia-lyase (*PAL*) were more strongly upregulated in leaves than in roots. For *PAL*, a fold change of 5.0 was detected in leaf tissue, whereas a fold change of 2.1 was found in roots (**Figure 3**). Fold changes for *JMT* were 4.8 in leaf tissue and 3.6 in roots. However, the difference in the expression level between samples from ARD soil and γARD soil was not significant for roots (*p* = 0.21) due to higher variability.

### Expression of CGs in Response to Different Soil Origins

**Figure 4:**
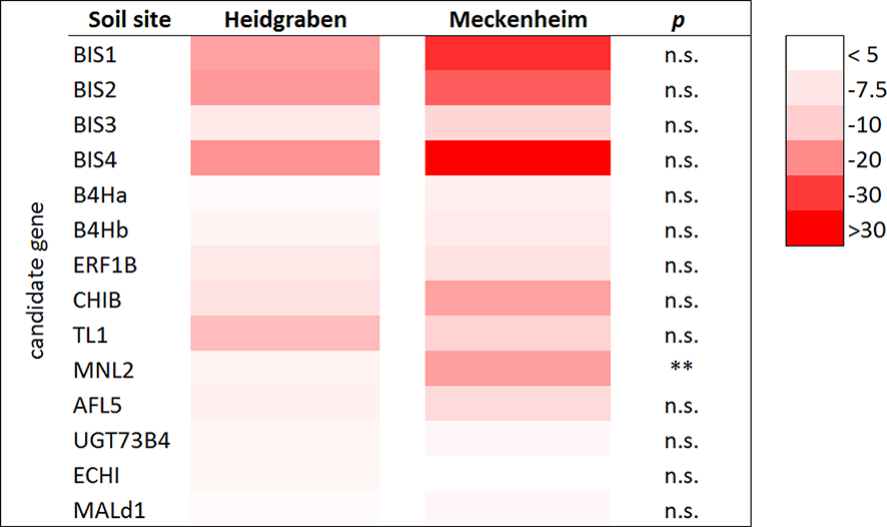
Regulation of CG expression in roots of plants grown in the ARD soils Heidgraben and Meckenheim including mean gene expression data of the genotypes B63, M26, and MAL0595, with five replicates for each genotype. The heat map indicates the fold change values (ARD soil/γARD soil) with the rows displaying the selected 14 CGs with an average significant fold change value > 4.5 in root tissue. The columns display the soil sites Heidgraben and Meckenheim. The intensity of the red color corresponds with the detected fold change value. *p* < 0.01 (**) and n.s., not significant.

The 14 CGs with a significantly increased expression in roots were compared in plants grown in Meckenheim soil and in Heidgraben soil. As no data for M9 were available for Meckenheim soil, only B63, M26, and MAL0595 were considered for this comparison. Although differences in gene expression between the two soil types were found for all genes with a stronger upregulation in the soil from Meckenheim, the overall differences (including data of all genotypes) were mostly not statistically significant. The only exception is *MNL2*. This gene was expressed at a significantly higher level (3.2 times) in Meckenheim soil (18.4-fold to 5.7-fold, *p* < 0.05) (**Figure 4**).

### Genotypic Differences in CG Expression

**Figure 5:**
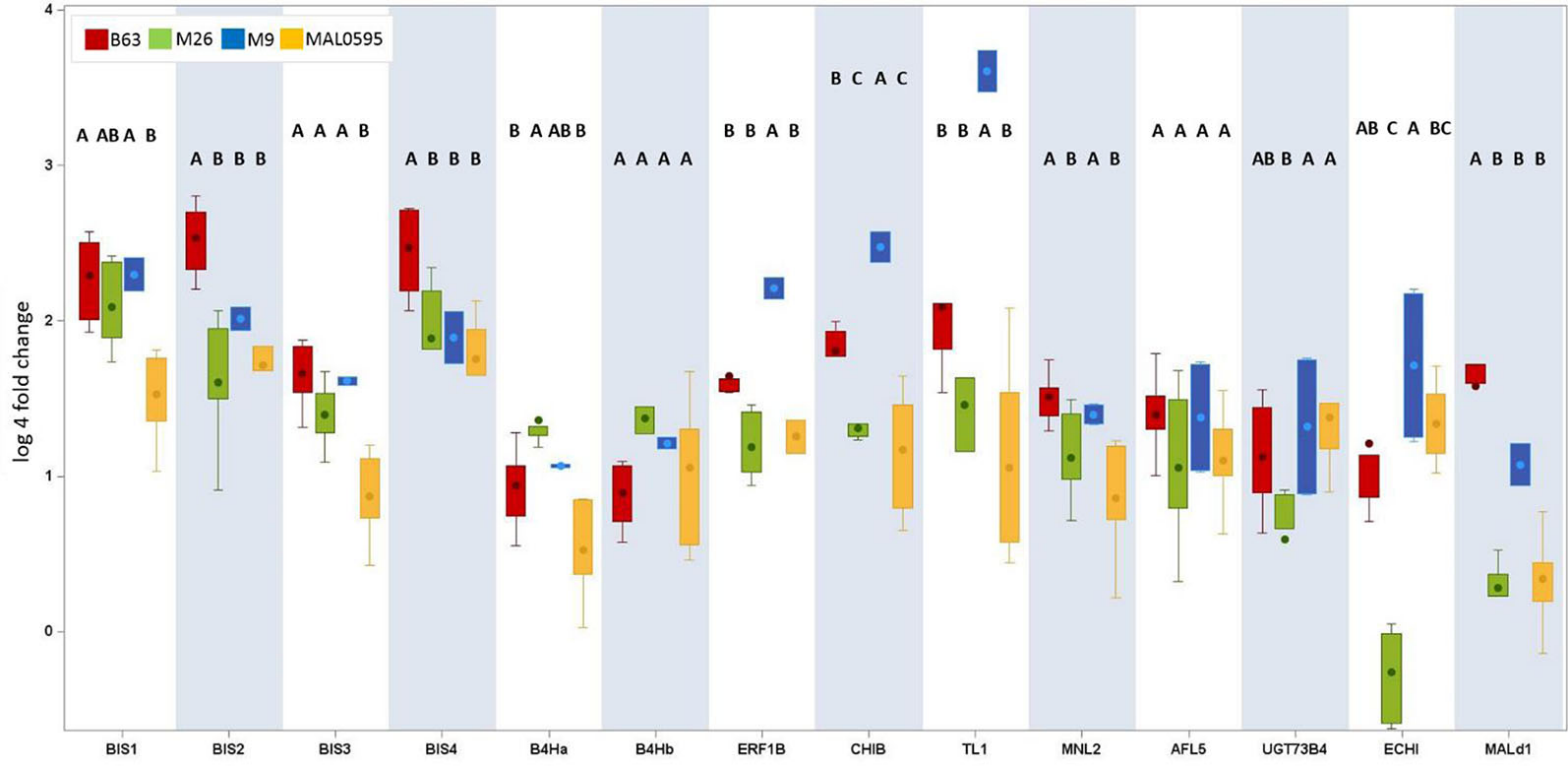
Genotypic differences in the regulation of the 14 CGs. This box plot presents only CGs with an upregulation (fold change ARD soil/γARD soil > 4.5) in root tissue. The root-specific average fold change values of all genotypes including five (B63, M26, MAL0595) or two replicates (M9), respectively, of 14 genes after cultivation in Heidgraben ARD soil are shown. The letters denote the significant differences between the genotypes for one gene. Significant differences are indicated by different letters. The whiskers were not drawn to the minimum or maximum values, if they were longer than 1.5 times the IQR. Data points outside of this range of 1.5 × IQR were indicated as outliers (dots).

Genotypic differences in the expression of the 14 CGs were studied for all four genotypes after cultivation of plants in Heidgraben soil (**Figure 5**). *BIS1, BIS2, BIS3*, and *BIS4* were upregulated in all genotypes, except *BIS3* in MAL0595. The highest increase was found in B63, the lowest in MAL0595. The differences between these two genotypes were statistically significant. For *BIS2* and *BIS4*, significant differences were found between B63 and the three genotypes M26, M9, and MAL0595. The upregulation of *BIS1* and *BIS3* was comparable for B63, M9, and M26, but significantly lower in MAL0595. The least differences were found for *B4Ha* and *B4Hb*. Significant differences were detected only between B63 and M26, for *B4Ha*.

*MNL2* showed the highest upregulation in B63 and the lowest in MAL0595. Statistically significant differences were also detected between B63/M9 and M26,/MAL0595. Genotypic differences were also found for *CHIB, ERF1B*, and *TL1*. For *ERF1B*, the fold changes were highest in M9 and lowest in M26,. For *CHIB* and *TL1*, the fold changes were highest in M9 and lowest in MAL0595. Differences were statistically significant between M9 and the other genotypes (**Figure 5**).

For *ECHI,* statistically significant differences were found between M26, (lowest regulation) and M9 (highest regulation). *Mal d1* showed highest upregulation in B63 with statistically significant differences to M26, M9, and MAL0595. The fold change of *UGT73B4* was less pronounced. Nevertheless, the detected differences were statistically significant between M26, and MAL0595 and between M26, and M9. No genotype-specific differences were found for *AFL5*.

### Phytoalexin Biosynthesis in Roots

**Figure 6:**
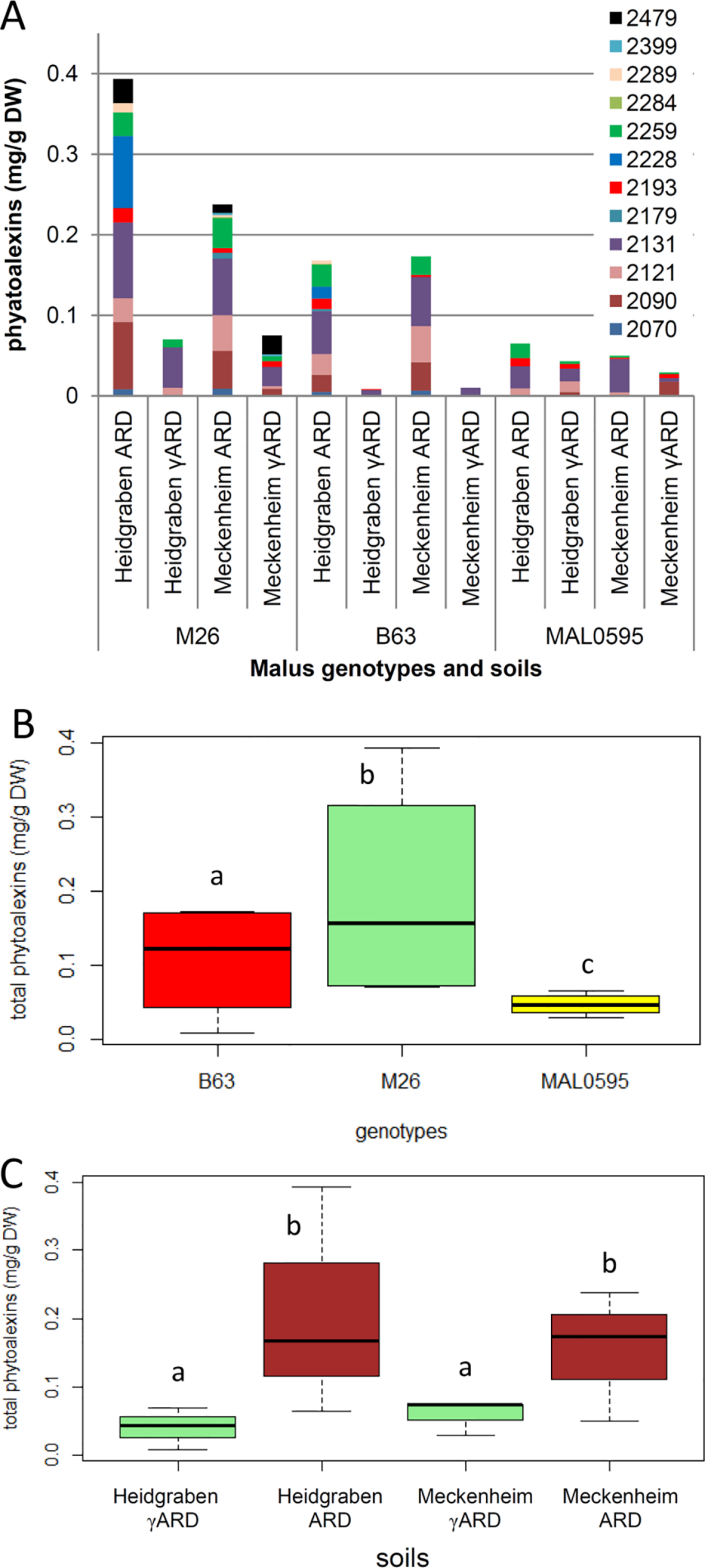
Analysis of phytoalexins in roots of the three genotypes M26, B63, and MAL0595, which were grown for 4 weeks on ARD and γARD soils from the two sites Heidgraben and Meckenheim. **(A)** Levels of individual phytoalexins identified by gas chromatography–mass spectrometry (GC-MS). Compound retention index (RI) 2,070, isomer of noraucuparin; 2,090, aucuparin; 2,121, noraucuparin; 2,131, 2-hydroxy-4-methoxydibenzofuran; 2,179, isomer of eribofuran; 2,193, 2'-hydroxyaucuparin; 2,228, eribofuran; 2,259, noreriobofuran; 2,284, isomer of hydroxyeribofuran; 2,289, isomer of noreriobofuran; 2,399, methoxyeribofuran; 2,479, 3,9-dimethoxy-2,4-dihydroxydibenzofuran. **(B, C)** Total phytoalexin content as a function of genotype and soil, respectively. Different letters indicate significant differences revealed by Tukey test (n = 8 for B and n = 6 for C) applied to the total phytoalexins.

A total of 12 biphenyl and dibenzofuran phytoalexins were detected and quantified in the roots of the *Malus* rootstock genotypes M26, B63, and MAL0595, which were grown in the two different soils for 4 weeks (**Figure 6A**). Significant differences in phytoalexin production were observed among the genotypes. M26, roots contained the highest phytoalexin amount and MAL0595 had the lowest, while B63 had an intermediate level of phytoalexins (**Figure 6B**). Furthermore, MAL0595 formed only three biphenyls and two dibenzofurans, whereas M26 and B63 produced the majority of the four biphenyls and eight dibenzofurans analyzed (**Table S1**). Notably, phytoalexin biosynthesis was significantly induced by ARD soils from both sites, whereas the difference in total phytoalexin content between the two soil sites was not significant (**Figure 6C**). Among the five main phytoalexins detected, the amount of 2-hydroxy-4-methoxydibenzofuran with a retention index (RI) of 2,131 was the highest. It was the only compound that was observed in all the samples including those from γARD soils. The content of 2-hydroxy-4-methoxydibenzofuran was upregulated by the ARD soils in all genotypes. The same held true for the other four major phytoalexins (RI 2,090; 2,121; 2,228, and 2259, respectively), except for aucuparin (RI 2,090), which was downregulated in MAL0595 in both soil types (**Table S1**). Another phytoalexin, 2'-hydroxyaucuparin (RI 2,193), also showed an interesting soil-dependent regulation pattern. In Heidgraben ARD soil, its content was upregulated in all genotypes; however, in Meckenheim ARD soil its content was downregulated (**Table S1**). However, differences between the three genotypes in their responses to the two soil sites were also observed. In MAL0595 roots, the formation of new phytoalexin compounds was not induced by ARD soil from both sites, while the formation of five and eight new phytoalexins was induced by ARD soil in M26, and B63 roots, respectively. Furthermore, M26 and B63 formed only two and four phytoalexin compounds, respectively, when grown in Heidgraben γARD soil, but produced eight phytoalexin compounds each in Meckenheim γARD soil (**Figure S1**).

### Correlation Between Phenotypic Data, Gene Expression Data, and Phytoalexin Contents

A Pearson's correlation was calculated between gene expression (fold changes of the 14 CGs expressed in root tissue) and changes in biomass and shoot length. The highest correlation was found between *CHIB* expression and shoot length (*r *= 0.96; *p* < 0.01). Highly significant correlations were also found between *B4Ha, BIS1, BIS3,* and *BIS4* expression and shoot length (**Table 3**). A statistically significant correlation between the biomass and the expression of any of the CGs was not observed.

**Table 3:** Pearson’s correlation between the differences in the expression of the 14 candidate genes (CGs, expressed in fold changes) and the differences in fresh biomass and shoot length of plants grown in ARD soil compared to those grown in γARD soil (biomass/ shoot length in ARD soil - biomass/ shoot length in γARD soil), significant correlations are given in bold with p < 0.05 (*), p < 0.01 (**), and p < 0.001 (***).

CG	Biomass^1^	Shoot length^1^
*BIS1*	−0.57	−0.92**
*BIS2*	−0.26	−0.65
*BIS3*	−0.46	−0.86*
*BIS4*	−0.57	−0.91**
*B4Ha*	−0.41	−0.82*
*B4Hb*	−0.31	−0.77
*ERF1B*	−0.38	−0.49
*CHIB*	−0.67	−0.96***
*TL1*	−0.23	−0.15
*MNL2*	−0.11	−0.49
*AFL5*	0.21	0.39
*UGT73B4*	−0.44	−0.21
*ECHI*	0.01	0.11
*Mal d1*	−0.44	−0.69

1 Measured after 28 days of cultivation in the greenhouse.

Statistically significant correlations were also found between some phytoalexin compounds and the changes in expression of the six CGs belonging to the biphenyl biosynthesis pathway. The amount of noraucuparin was most strongly correlated with the expression of *B4Ha*, *BIS1*, *BIS3*, and *BIS4* (*r* = 0.70 to 0.73; *p* = 0.01, **Table 4**). *B4Ha* expression was correlated with the amount of 2-hydroxy-4-methoxydibenzofuran and the isomer of noraucuparin. A significant correlation was found between the total amount of phytoalexins and the changes in expression of *B4Ha* (*r* = 0.60; *p* = 0.04).

**Table 4:** Pearson's correlation between changes in the expression of the six CGs of the phytoalexin biosynthesis pathway (expressed as fold changes) and the amounts of individual phytoalexins, *p* < 0.05 (*) and *p* < 0.01 (**). Intensity of red shading visualizes strength of correlation. Significant correlations are given in bold.

Phytoalexins	Candidate gene											
	BIS1		BIS2		BIS3		BIS4		B4Ha		B4Hb
2-hydroxy-4-methoxydibenzofuran	0.52		0.45		0.54		0.51		**0.65**	*	**0.58**
Aucuparin	0.40		0.20		0.41		0.33		0.56		0.47
Isomer of noraucuparin	**0.57**		0.41		**0.57**		0.54		**0.64**	*	0.54
Noraucuparin	**0.73**	**	0.55		**0.71**	**	**0.70**	**	**0.73**	**	0.67
Noreriobofuran	0.49		0.40		0.51		0.49		0.52		0.49
Phytoalexins total	0.44		0.29		0.46		0.38		0.60	*	0.53

### Protein–Protein Interaction Analysis

Accession numbers of 17 DEGs with a significant fold change > 1.5 in roots were integrated into a protein interaction network using TAIR (The Arabidopsis Information Resource, 6). These proteins included the highly expressed *BIS* (fold change > 20.0), *CHIB* and *MNL2* (fold change > 10.0), as well as *ERF1B, B4H*, and *PAL* (fold change > 4.5). For the remaining 22 DEGs, no interaction was found.

**Figure 7:**
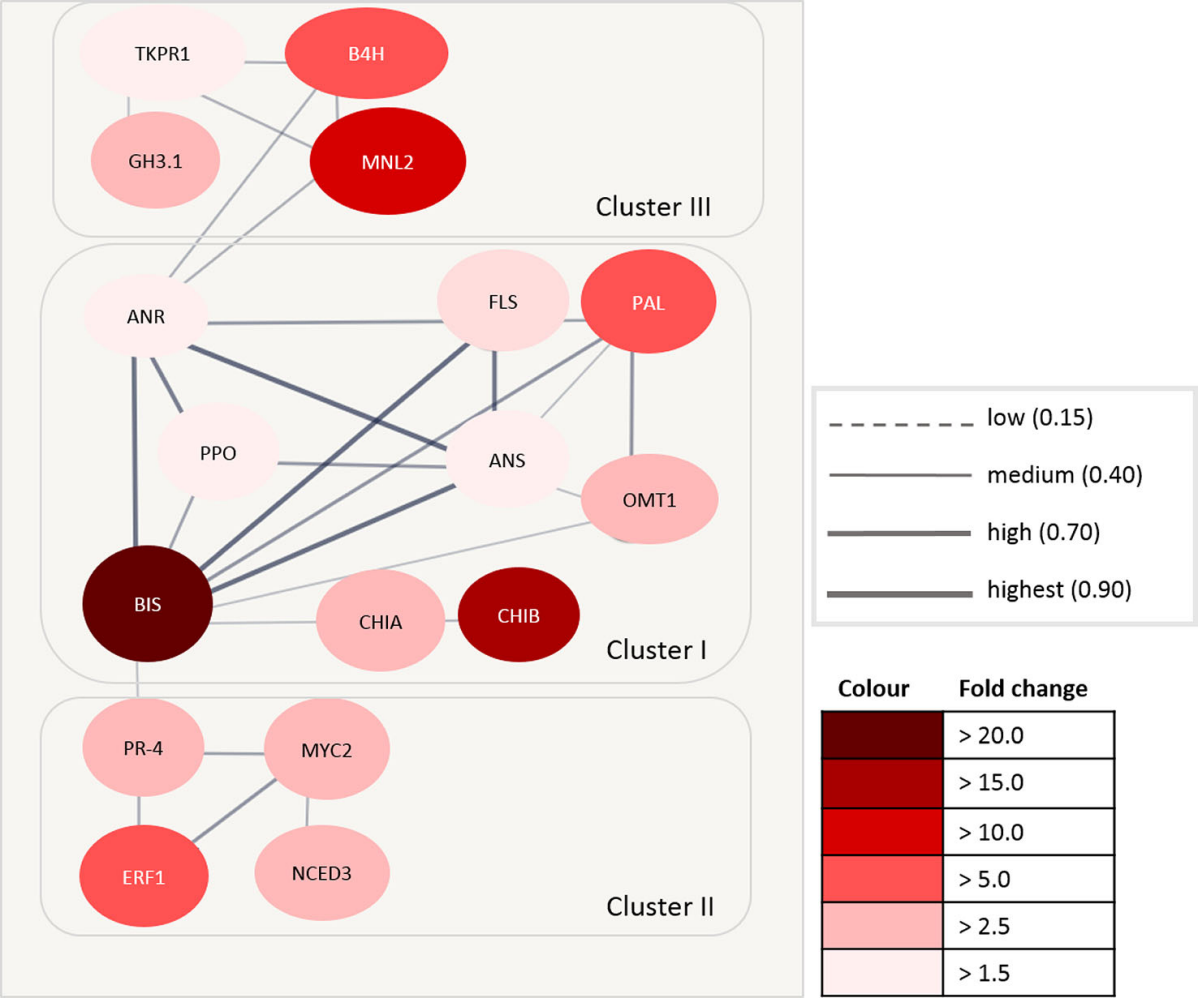
Protein–protein interaction matrix using The Arabidopsis Information Resource (TAIR) accession number of differentially expressed genes (DEGs) in *Malus*. The circles represent the proteins, and the lines between circles represent the interaction between individual proteins. The thickness of the lines defines the confidence of the interaction. The intensity of the red color indicates the fold change expression value (ARD soil/γARD soil). *ANR*, anthocyanidin reductase; *ANS*, anthocyanidin synthase; *B4H*, biphenyl 4-hydroxylase; *BIS*, biphenyl synthase; *CHIA*, acidic endochitinase-like; *CHIB*, endochitinase EP3-like; *ERF1*, ethylene-responsive transcription factor 1; *FLS*, flavanone 3-hydroxylase; *GH3.1*, indole-3-acetic acid-amido synthetase; *MNL2*, putative mannosidase; *NCED3*, nine-cis-epoxycarotenoid dioxygenase 3; *TKPR1*, tetraketide alpha-pyrone reductase 1; *MYC2*, transcription factor MYC2; *OMT1*, O-methyltransferase 1; *PAL*, phenylalanine ammonia-lyase; *PPO*, polyphenol oxidase; *PR-4*, pathogenesis-related protein PR-4.

The highest confidence of a protein–protein association was found in the first network cluster (**Figure 7**). This cluster consisted of *BIS*, *CHIA*, *O*-methyltransferase 1 (*OMT1*), polyphenol oxidase (*PPO*), *PAL*, anthocyanidin reductase (*ANR*), and anthocyanidin synthase (*ANS*). Two further proteins within this cluster are involved in the signal transduction process (*CHIB*) and the oxidation–reduction process (flavanone 3-hydroxylase, *FLS*). *BIS* showed the highest confidence of interaction with *ANR, ANS,* and *FLS*. The confidence of interaction of *BIS* with other proteins within this cluster was medium to high.

The second cluster was comprised of four proteins with a medium to high confidence of interaction. Three proteins (*ERF1B*, transcription factor *MYC2,* pathogenesis-related protein *PR-4*) were involved in the signaling pathway, whereas one seems to be involved in abscisic acid biosynthesis (nine-*cis*-epoxycarotenoid dioxygenase 3, *NCED3*).

The third cluster contained four proteins of different functions. These proteins were grouped with a low to medium confidence of interaction. *B4H* is involved in phytoalexin biosynthesis, whereas tetracetide *alpha*-pyrone reductase 1 (*TKPR1*) belongs to the brassinosteroid biosynthesis pathway. Indole-3-acetic acid-amido synthetase (*GH3.1*) belongs to the auxin biosynthesis pathway. The function of *MNL2* is unknown, but it is associated to the oxidoreductase family.

## Discussion

### Phytoalexin Biosynthesis Is Strongly Increased in Response to ARD

It is generally accepted that ARD is strongly associated with an unbalanced complex of soil biota, including bacteria, fungi, oomycetes, and nematodes (Rumberger et al., 2007; Kanfra et al., 2018). In the present study, the expression changes of 108 ARD CGs were evaluated in roots of three different *Malus* rootstocks and one wild apple genotype grown in ARD soils from two different sites in Germany. The most highly upregulated CGs in ARD soil were genes related to the phytoalexin biosynthesis, including the four biphenyl synthase genes *BIS1*, *BIS2*, *BIS3*, and *BIS4* and the two biphenyl 4-hydroxylase genes *B4Ha* and *B4Hb* (**Figure 4**). *BIS* and *B4H* genes encode for enzymes involved in the biosynthesis of biphenyl and dibenzofuran phytoalexins (**Figure S2**). These phytoalexins are only formed by plants belonging to the subtribe Malinae of the family Rosaceae, such as members of the genera *Malus* and *Pyrus* (Liu et al., 2007; Beerhues and Liu, 2009; Liu et al., 2011; Chizzali and Beerhues, 2012; Sircar et al., 2015). The results of CG expression correlated well with the total phytoalexin content, which was also significantly increased in the roots of plants grown in ARD soils (**Table 4**, **Figure 5C**). It has to be mentioned here, that the roots were sampled 3 weeks earlier for gene expression analyses than for phytoalexin detection, because after 1 week of culture, the amount of root fresh mass was not sufficient to enable both kinds of analyses. Moreover, the culturing period of 4 weeks was necessary to record the biomass data that allowed a clear classification of the soils as ARD soils based on the observed growth depression.

Comparable results for the expression of these CGs genes were also obtained in other studies on apple, either in response to the necrotrophic pathogen *P. ultinum* or in response to ARD soil (Zhu et al., 2014; Weiß et al., 2017a; Weiß et al., 2017b; Zhu et al., 2017). Phytoalexins are part of the complex defense system of plants against pests and pathogens (Jeandet et al., 2014). The induction of phytoalexin biosynthesis seems to be one of the induced defense responses of *Malus* rootstocks to stresses caused by the biota in ARD soils. The antifungal and antibacterial activities of biphenyls and dibenzofurans was clearly shown although their precise mechanisms of action are still unknown (Chizzali and Beerhues, 2012). Loss-of-function experiments on other plant–pathogen interactions have demonstrated that reduced levels of phytoalexins lead to increased disease susceptibility (Jeandet et al., 2014). Examples are known from pea (Wu and VanEtten, 2004), soybean (Graham et al., 2007), sorghum (Ibraheem et al., 2010), pear (Chizzali et al., 2016), and *Arabidopsis* (Jeandet et al., 2013). However, there are also reports that high phytoalexin concentrations may be toxic to plant cells (Dixon et al., 1994; Rogers et al., 1996), which was also hypothesized by Weiß et al. (2017b) for apple rootstocks. The accumulation of high concentrations of phytoalexins in ARD-susceptible rootstocks like M26 and B63 may cause root damage and even death. It was assumed that the exudation mechanism or the detoxification system do not work properly in these genotypes. This hypothesis is supported by the results obtained with the less susceptible genotype MAL0595. This genotype accumulated significantly less phytoalexins in roots compared to M26 and B63. Consistently, the reduction in shoot length of MAL0595 plants grown in ARD soils was not statistically significant (**Figure 6C**, **Table 2**).

Among the four *BIS* genes, the highest upregulation in ARD soils was observed for *BIS1*, followed by *BIS2* and *BIS4*. However, *BIS3* transcript level exceeded the transcript levels of the other *BIS* genes in the roots by far (**Table S5**). As previously reported by Chizzali et al. (2012a) and other authors, the regulation of the individual *BIS* genes can differ depending on the pathogen and the type of the infected tissue. In a transcriptome analysis conducted with M26 grown in ARD soil, the expression of *BIS2*, *BIS3*, and *BIS4* was induced, with *BIS3* showing the highest increase in roots (Weiß et al., 2017a). In the present study, *BIS3* expressional levels also were the highest (**Table S5**) but with the lowest fold change among the *BIS* genes investigated in the roots. Due to its exceedingly high expression level, *BIS3* seems to play a pronounced role in phytoalexin biosynthesis. *BIS4* showed the highest differences between the two soil types. After fire blight infection, *BIS1* and *BIS2* were upregulated in leaf tissue. In contrast, *BIS3* was strongly expressed in the stem, where it was spatially limited to the transition zone between healthy and necrotic tissue (Chizzali et al., 2016). In the present study, expression of *BIS* genes was also focused on the region affected by the biotic stress, the roots.

### Further CGs Involved in Biotic Stress Responses Are Upregulated

Primer efficiencies were calculated in a different experiment with a different PCR system for validation. In the Fluidigm system, the software calculates a quality score for each individual amplification curve by comparing the amplification curve to an ideal exponential curve. If the curve is close to the ideal one, the quality score approaches 1. The software sets a cutoff for the quality score of > 0.65 to exclude primers with poor efficiencies. Nevertheless, all data used were still without any PCR efficiency correction. Therefore, we decided not to consider smaller differences in gene expression, but focused on the CGs with fold changes above 4.5.

Among these, *TL1* and *Mal d1* were upregulated in roots after cultivation of plants in ARD soil. Similar results were obtained by Weiß et al. (2017a). The *TL1* product belongs to a highly complex protein family with antimicrobial and antifungal activities (Liu et al., 2010; Singh et al., 2013). Overexpression of *TL*s in transgenic wheat plants mediated enhanced resistance and protection against different fungal pathogens (Mackintosh et al., 2007). Mal d1 is a defense protein, which belongs to group 10 of pathogenesis-related proteins. It is expressed by plants in response to different stress conditions, such as pathogen infection, exposure to certain chemicals, wounding, and stressful environmental conditions (Puehringer, 2003). In apple fruits, Mal d1 is known as a birch pollen–related food allergen. Previous studies by our research group have shown that the synthesis is strongly related to exogenous stress factors (Schmitz-Eiberger and Matthes, 2011; Kiewning and Schmitz-Eiberger, 2013). However, its function in response to ARD remains to be elucidated.

*ERF1B*, *CHIB*, and *ECHI* also showed a notable fold change in root samples. *ERF1B* encodes a transcription factor that is involved in ethylene signaling. An *ERF1B*-mediated ARD defense response in apple roots was also observed in other studies (Shin et al., 2014; Weiß et al., 2017a). Ethylene is an essential mediator of biotic and abiotic stress responses (Müller and Munné-Bosch, 2015), and ethylene-responsive transcription factors (*ERF*) regulate the molecular response to pathogen attack (Ito et al., 2014; Müller and Munné-Bosch, 2015; Huang et al., 2016). Within the ethylene-mediated transcriptional response, the promoter region of *CHIB* may be a target of *ERF* transcription factors. Based on the results obtained, it could be assumed that the changes in *ERF* expression have led to a subsequent activation of *CHIB* (Shin et al., 2014). For other genes like *ACS* and *ACO*, which encode key enzymes of the ethylene biosynthesis, no upregulation was observed. It is common knowledge that different isoforms within a gene family can carry out specific functions in different plant processes (Shin et al., 2014). An involvement of other isoforms of *ACS* and *ACO*, which were not investigated in this study, cannot be excluded. The endochitinase EP3-like gene *CHIB* belongs to a large family of plant chitinase genes and is generally induced by pathogen attack and other biotic stresses (Hamid et al., 2013; Nagpure et al., 2014). Chitinases play a role in the biocontrol of fungal phytopathogens and plant defense systems especially against chitin-containing pathogens (Hamid et al., 2013).

The genes *MNL2, ALF5, ECHI*, and *UGT73B4* were also significantly upregulated in roots. These genes appear to be related to ARD, but have not been described in this context before. The putative mannosidase gene *MNL2* belongs to the glucose–methanol–choline oxidoreductase family. Genes of this family are involved in adaptive processes in plant–insect interactions during host-dependent chemical defense (Rahfeld et al., 2014). However, the detailed function of the *MNL2* gene in plants is still unknown. The *ALF5* gene belonging to the MATE gene family is expressed in root epidermis cells and is necessary for protecting roots from toxic compounds in the soil (Diener et al., 2001). Some genes within the MATE gene family are supposedly involved in transporting toxic compounds to infected parts of the plant in order to attenuate pathogen attack (Santos et al., 2017). Within the multigene family of plant UGTs, an upregulation was observed for *UGT73B4*. Plant glycosyltransferases usually use UDP-glucose in the transfer reactions catalyzed. Furthermore, it is assumed that UGTs are part of stress responses (Li et al., 2001; Langlois-Meurinne et al., 2005). Analysis of *A. thaliana* defense-signaling mutants indicated that expression of the corresponding UGT genes is necessary during the hypersensitive response (Dare et al., 2017). These results emphasize the importance of UGTs in plant–pathogen interactions (Dare et al., 2017). It is tempting to speculate that UGTs may be involved in the detoxification of biphenyl and dibenzofuran phytoalexins via glycosylation and deposition in the central vacuole. However, no glycosylated derivatives of the defense compounds have so far been detected in infected plants and elicitor-treated cell cultures of the Malinae, except for two glucosides (aucuparin and eriobofuran derivatives), which were isolated from cell cultures of the scab-resistant apple cultivar ‘Liberty’ (Borejsza-Wysocki et al., 1999). Because of their general function in pathogen defense, *ALF5*, *ECHI*, and *UGT73B4* appear to also be activated by pathogens of the ARD complex. However, further investigations will be necessary to elucidate their precise function.

### Two CGs Showed Upregulation in Leaf Tissues

*PAL* and *JMT* showed a stronger upregulation in leaf tissue than in roots. The *PAL* gene encodes for the enzyme phenylalanine ammonia-lyase, which is the key enzyme of the phenylpropanoid pathway. Repression of this pathway in apple via a reduction in key transcript levels (e.g. for *PAL*), and enzyme activities (e.g. PAL and chalcone synthase) resulted in severe dwarfing and internode length reduction (Dare et al., 2017). The occurrence of stunted shoots because of ARD infection seems therefore to be independent of the *PAL* gene expression level. Whether shoot stunting is connected to the occurrence or the amount of individual phenolic compounds or not remains to be investigated. The *JMT* gene encodes for the enzyme S-adenosyl-l-methionine:jasmonic acid carboxyl methyltransferase (*JMT*), which catalyzes the formation of methyl jasmonate from jasmonic acid. Plants produce jasmonic acid and methyl jasmonate in response to many biotic and abiotic stresses, in particular, herbivory and wounding (Seo et al., 2001; Wasternack, 2007). Both genes (*PAL* and *JMT*) are associated with pathogen defense reactions and stress response. The upregulation of their expression in leaf tissue could be an indication for biotic stress because of ARD infection. However, their precise role in connection with ARD has to be further investigated.

### The Soil Origin Influences Plant Growth, CG Expression and Phytoalexin Production

The expression of CGs was compared between plants grown in Meckenheim soil and Heidgraben soil. The genes *MNL2, BIS1, BIS2*, *BIS4*, and *TL1* showed a strong upregulation in roots of all genotypes if plants were grown in ARD soil. This was the case for both soil types, although the upregulation was more pronounced in plants grown in Meckenheim soil (**Figure 4**). Even though a stronger increase in gene expression was observed, the total phytoalexin amount was not increased in plants of Meckenheim soil. The differences found between the two soils were not statistically significant (**Figure 6C**). It is assumed that each genotype seems to produce phytoalexins up to a certain level, once the biosynthesis is stimulated by ARD soil. So far, the rate-limiting steps of biphenyl and dibenzofuran biosynthesis remain unknown. Compared to the *BIS* genes, the fold changes in the expression of *B4Ha* and *B4Hb* were markedly lower, and genes for *O*-methyltransferases (Khalil et al., 2015) were not among the upregulated genes. Due to the incomplete examination of the phytoalexin biosynthetic pathway, some genes remain to be identified, including the gene coding for the enzyme that converts aucuparin to 2'-hydroxyaucuparin (**Figure S2**). This gene should be highly expressed in ARD soil from Heidgraben, because the 2'-hydroxyaucuparin content of roots grown in this soil was greatly increased compared to that of roots from γARD soil. In roots of plants grown in ARD soil from Meckenheim, the reaction of this gene might be different; the expression of this gene does not seem not to be induced or even inhibited because of the decrease of the 2'-hydroxyaucuparin content in samples of these roots (**Figure S1**). Thus, different ARD soils may differently affect individual phytoalexin biosynthetic steps, leading to qualitative and/or quantitative changes in the phytoalexin patterns. Previously, varying phytoalexin patterns were observed in cell cultures of *Sorbus aucuparia* upon treatment with different elicitors, which, for example, stimulated the accumulation of aucuparin or eriobofuran as the major components (Hüttner et al., 2010). In the present study and a previous one (Weiß et al. 2017b), relatively high levels of 2-hydroxy-4-methoxydibenzofuran were even detected in roots from γARD soils. This indicates that the formation of this compound does not necessarily need the ARD biome stimulus although ARD soils lead to a further strong increase in the accumulation.

Soil properties can influence the extent of ARD directly or indirectly (von Bronsart, 1949; Franke-Whittle et al., 2018; Mahnkopp et al., 2018). The soil pH value is one of these properties, which has been discussed several times in this context. However, the results published about the effect of the soil pH value onto ARD severity were contrasting. In some cases, it was shown that a low soil pH seemed to be associated with a high degree of ARD (Willett et al., 1994; Mahnkopp et al., 2018). In other studies, it was found that ARD symptoms were less pronounced in soils with a low pH value (Jonkers et al., 1980; Utkhede et al., 1992). We found a higher overall fold change of CG expression on the silty soil of Meckenheim with a high pH (6.7) compared to the sandy soil (Heidgraben) with a lower pH value of 5.3. However, the effect of soil pH on ARD should not be overestimated. Changes in pH are not induced by apple replanting and are generally an unstable parameter (Mahnkopp et al., 2018). Different rootstocks seem to have a different growth optimum regarding the soil pH value. Some rootstocks achieve optimal growth at a low pH (e.g. CG.6589), whereas other rootstocks are well adapted to more calceous soils (e.g. CG41) (Fazio et al., 2012).

Soil organic matter (SOM) seems also to reduce the induction of ARD (Franke-Whittle et al., 2018). In our study, a remarkably lower SOC (soil organic carbon = total carbon due to absence of carbonate) of 12.3 g kg^−1^ in Meckenheim soil compared to a SOC of 25.4 g kg^−1^ in Heidgraben soil (**Table 1**), corresponded to a higher CG regulation. Plant growth in terms of shoot and root fresh biomass was significantly reduced on both soils for the sensitive genotypes M26, and B63 (**Table 2**). The only exception was root fresh mass of B63, which was not significantly reduced when grown on Meckenheim soil. These observations are interesting as they stress the limits of growth-based bio-tests to determine ARD severity. Factors like the high available water capacity of the loamy Meckenheim soil in comparison to the sandy Heidgraben soil may explain differences in plant growth besides ARD severity.

Soil biota like nematodes, which are part of the ARD complex, are also influenced by soil texture (Hbirkou et al., 2011). Is has been described that sandy soils are often more prone to ARD than loamy soils (Mahnkopp et al., 2018; Winkelmann et al., 2019). In this study, the upregulation of the CGs was less pronounced in the sandy Heidgraben soil compared to the silty Meckenheim soil, which is not in agreement with the findings mentioned above. Similar observations were made by Fazio et al. (2012) in a study investigating the influence of soil pH value and soil texture on ARD. Based on plant growth, some rootstocks appeared to be less sensitive to ARD in clay soil than in sandy soil, but also the opposite relationship was observed because other rootstock cultivars appeared to be more sensitive to ARD in the clay soil compared to the sandy soil (Fazio et al., 2012). These results indicate that soil properties cannot be judged without knowledge of the soil biome composition. Likewise, Mazzola and Manici (2012) concluded that abiotic factors may reduce or intensify ARD, but up to now, a causal relationship of a single abiotic factor and ARD is not evident.

Many soil characteristics influence plant growth. Therefore, growth-based bio-tests are limited in their information value regarding ARD severity. Additional methods for a more reliable diagnosis and possible quantification of ARD are of interest. Our data suggest that the expression of certain CGs may be a starting point for the identification of early indicators as an addition to growth data. To evaluate their usefulness in different ARD situations, especially under field conditions, further studies are necessary. These studies should also include the comparison to virgin soils on which no Rosaceae plants had been grown before and which are collected close to the replant sites.

### Genotypic Differences are Found for Gene Expression in Response to ARD

The four *Malus* genotypes M9, M26, B63, and MAL0595 used in our gene expression study possess susceptibility to ARD to different extents. M9 and M26, were previously classified as susceptible genotypes (Isutsa and Merwin, 2000; Leinfelder and Merwin, 2006; St Laurent et al., 2010). MAL0595 was grouped as a less sensitive genotype (Cummins and Aldwinckle, 1983; Reim et al., 2019). By contrast, no information regarding its ARD reaction was available for the rootstock genotype B63 at the beginning of our experiment. Recently, root microscopic and bio-test data proved this genotype to react similarly to M26, in response to ARD (Grunewaldt-Stöcker et al., 2019). Our present results on plant growth and CG expression support the observation that B63 has to be considered as ARD-sensitive.

In the present study, the genotype-specific gene expression data (**Figure 5**) are consistent with the phytoalexin detection results (**Figure 6B**). Relatively low gene expression levels yield a relatively low total phytoalexin content in MAL0595, whereas high gene expression levels lead to high phytoalexin contents in M26, and B63 (**Figures 4** and **5C**). Besides the quantitative differences, there was also qualitative variation in the phytoalexin patterns. In γARD soils, roots of MAL0595 formed few phytoalexins at low levels, which increased partly upon growth in ARD soils from the two sites. While the 2-hydroxy-4-methoxydibenzofuran content was strongly enhanced, the noraucuparin content decreased (**Figure S1**). No new phytoalexins were formed. In M26, and B63, the aucuparin, noraucuparin, and noreriobofuran levels increased greatly in both ARD soils. Therefore, these three phytoalexins may be the major compounds that cause cytotoxicity to apple roots. For the growth data, the results for MAL0595 were opposite to those for M26, and B63. Thus, the low fold changes of most CGs in response to ARD (**Figure 6**), the low phytoalexin content compared to the other genotypes (**Figures 5** and **S1**), and the low growth depression (**Table 2**) match the classification by phenotypic data of MAL0595 as less susceptible to ARD. The assessment of these parameters may similarly provide information about the degree of ARD susceptibility of other genotypes. Further studies should clarify if the *BIS* genes can be used as possible biomarkers for ARD susceptibility, as their expression correlated well with the observed susceptibility as classified on phenotypic data. *BIS3* seems to play a key role in phytoalexin biosynthesis under replant conditions, because *BIS3* transcript levels exceeded the other *BIS* genes by far, but overall they were regulated less strongly.

## Conclusions

The expression of 108 CGs was studied in leaf and root tissue of four different *Malus* genotypes grown in ARD soil and γARD soil from two different sites in Germany. For most of these genes, it is the first time that their tissue specific expression pattern was investigated in different genotypes and in response to ARD. The data obtained allow conclusions about general (genotype-independent) and genotype-dependent effects of ARD on the expression of these genes. Changes in CG expression were more frequent and more pronounced in root tissue compared to leaf tissue. This result suggests that the response of *Malus* plants to ARD is local. The defense reaction seems to be spatially restricted to the site of infection. A systemically acquired stress response could not be detected. Sixteen CGs were strongly upregulated in roots of plants grown in ARD soil. Six of them belong to the phytoalexin biosynthesis pathway. Their expression patterns were consistent with the phytoalexin content. It can be assumed that phytoalexins may play a role in the reaction of *Malus* plants to ARD. However, their function in the disease etiology remains to be clarified. The expression patterns of the biphenyl synthase genes *BIS1*, *BIS2 BIS3*, and *BIS4* correlated well with the phenotypic reaction of the *Malus* genotypes investigated with *BIS3* showing the strikingly highest normalized expression. These genes are useful as biomarkers to identify the presence of ARD inducing microbiota in unknown soil samples. They may also give clear indications for the defense reaction of plants growing at a site, whose state with regard to ARD is unknown.

## Data Availability Statement

The datasets generated for this study can be found in the Gene Expression Omnibus https://www.ncbi.nlm.nih.gov/geo/query/acc.cgi?acc=GSE135081. Data from the greenhouse experiment is deposited in the BonaRes Data Center (DOI: 10.20387/bonares-R1S0-52TX).

## Author Contributions

SR and A-DR were involved in performing the experiments, analyzing the data, and writing the manuscript. SW and MS contributed to CG selection and writing the manuscript. BL and LB conceived and designed the phytoalexin experiments. BL and A-DR analyzed the phytoalexin compounds in root material and interpreted the data. HF, M-VH, and TW conceived and coordinated the project, and participated in data interpretation and writing the manuscript.

## Funding

This work was part of the project BonaRes-ORDIAmur funded by the German Federal Ministry of Research and Education within the frame of the program BonaRes (grant no. 031B0025B). It was also funded by the German Research Foundation (DFG) via the research training group GRK1798 “Signaling at the Plant-Soil Interface” and a grant to BL and LB (BE 1174/19-1).

## Conflict of Interest

The authors declare that the research was conducted in the absence of any commercial or financial relationships that could be construed as a potential conflict of interest.
