# Corrigendum: Genes Involved in Stress Response and Especially in Phytoalexin Biosynthesis Are Upregulated in Four *Malus* Genotypes in Response to Apple Replant Disease

**DOI:** 10.3389/fpls.2021.723957

**Published:** 2021-07-28

**Authors:** Stefanie Reim, Annmarie-Deetja Rohr, Traud Winkelmann, Stefan Weiß, Benye Liu, Ludger Beerhues, Michaela Schmitz, Magda-Viola Hanke, Henryk Flachowsky

**Affiliations:** ^1^Institute for Breeding Research on Fruit Crops, Julius Kühn-Institut, Federal Research Centre for Cultivated Plants, Dresden, Germany; ^2^Institute of Horticultural Production Systems, Woody Plant and Propagation Physiology Section, Gottfried Wilhelm Leibniz University Hannover, Hanover, Germany; ^3^Institute of Pharmaceutical Biology, Technische Universität Braunschweig, Braunschweig, Germany; ^4^Department of Natural Sciences, Hochschule Bonn-Rhein-Sieg, Rheinbach, Germany

**Keywords:** apple replant disease (ARD), gene expression, BioMark HD microfluidic system, high-throughput qRT-PCR, phytoalexins, greenhouse bio-test, soil properties, *Malus* genotypes

In the original article, there was a mistake in Figure S2 as published. The mistake included the labeling of two steps in the proposed biosynthesis pathway of biphenyl and dibenzofuran phytoalexins. The corrected Figure S2 appears below. Figure S2 has been updated in the original article.

**Figure S2:**
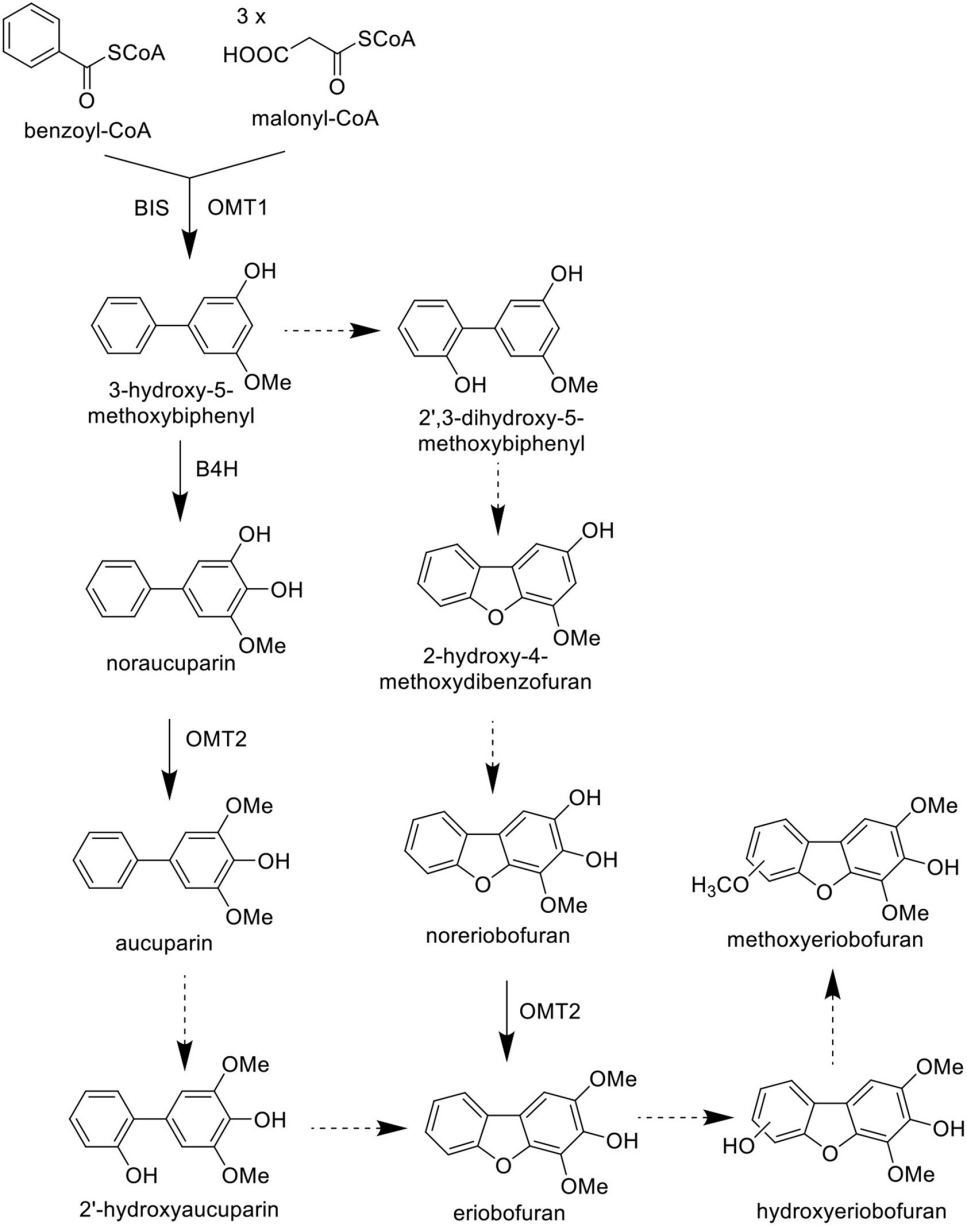
Proposed biosynthetic pathway of biphenyl and dibenzofuran phytoalexins. Solid arrows represent established steps whereas broken arrows mark hypothetical reactions. BIS, biphenyl synthase; OMT, *o*-methyltransferase; B4H, biphenyl 4-hydroxylase.

In **Table S2** of the published article, single amplicon sizes were given incorrectly. They have been corrected, and a corrected **Table S2** file is published.

Following a reader's comment, specificity of two primer pairs was tested by sequencing. A paragraph was added to **Material And Methods**, **Primer Selection and RT-qPCR Validation** after paragraph 2:

To test the specificity of the primers used to amplify the genes *B4Ha* and *B4Hb* (**Table S2**), an amplicon deep sequencing was conducted. The sequence analysis proved the *B4Hb* primers to be highly specific. The sequencing results also showed that the *B4Ha* amplicon is present in both *B4Ha* and *B4Hb*. This means that the primers for *B4Ha* are not gene-specific. This limited specificity should be considered for the interpretation of the respective data.

The authors apologize for these errors and state that they do not change the scientific conclusions of the article in any way. The original article has been updated.

## Publisher's Note

All claims expressed in this article are solely those of the authors and do not necessarily represent those of their affiliated organizations, or those of the publisher, the editors and the reviewers. Any product that may be evaluated in this article, or claim that may be made by its manufacturer, is not guaranteed or endorsed by the publisher.

