# Supplementary material for: Genes Involved in Stress Response and Especially in Phytoalexin Biosynthesis Are Upregulated in Four Malus Genotypes in Response to Apple Replant Disease
Source: Front Plant Sci. 2020 Feb 28;10:1724. doi: 10.3389/fpls.2019.01724 (PMC7059805; doi:10.3389/fpls.2019.01724)
Supplement: Supplementary file 1 [file DataSheet_1.pdf]

Figure S1: Detailed analysis of individual phytoalexins [µg/g DW] in roots of three genotypes grown for four weeks on ARD and γ-ARD soils from the two sites 'Heidgraben' (HG) and 'Meckenheim' (MH). The five major phytoalexins are highlighted in blue. The green and red arrows indicate genotype- and soil-specific, respectively, up- and downregulation of individual phytoalexins. PA, phytoalexin; MW, molecular weight; RI, retention index (RI) 2070, isomer of noraucuparin; 2090, aucuparin; 2121, noraucuparin; 2131, 2-hydroxy-4-methoxydibenzofuran; 2179, isomer of eribofuran; 2193, 2'-hydroxyaucuparin; 2228, eribofuran; 2259, noreriobofuran; 2284, isomer of hydroxyeribofuran; 2289, isomer of noreriobofuran; 2399, methoxyeribofuran; 2479, 3,9-dimethoxy- 2,4-dihydroxydibenzofuran.

Regulation		↑	↕	↑	↑	↑	↕	↑	↑	↑	↑	↓	↓		
Molecular weight (MW)		360	302	360	286	316	390	316	374	404	374	346	404	Total PA	No. of PA
Retention index (RI)		2070	2090	2121	2131	2179	2193	2228	2259	2284	2289	2399	2479		
B63	HG ARD	5	21↑	26	53	3	13↑	15	27	1	4	0	0	168	10
	HG γ ARD	0	0	0	7	0	1	0	0	0	0	0	0	9	2
	MH ARD	6	35↑	45	61	0	2	0	23	0	0	0	0	173	6
	MH γ ARD	1	8	3	24	0	↓7	0	6	0	0	2	23	75	8
M26	HG ARD	8	83↑	30	94	0	18↑	90	29	0	11	0	30	394	9
	HG γ ARD	1	0	9	50	0	0	0	10	0	0	0	0	70	4
	MH ARD	9	47↑	44	70	7	6	0	37	1	3	3	10	238	11
	MH γ ARD	1	8	3	24	0	↓7	0	6	0	0	2	23	75	8
MAL0595	HG ARD	0	0	9	27	0	10↑	0	18	0	0	0	0	65	4
	HG γ ARD	0	↓5	13	16	0	6	0	3	0	0	0	0	43	5
	MH ARD	1	0	3	42	0	1	0	3	0	0	0	0	50	5
	MH γ ARD	0	↓17	0	5	0	↓5	0	2	0	0	0	0	29	4
Total PA		33	224	187	473	10	77	104	165	2	18	8	88		
No. of samples containing the PA		8	8	10	12	2	11	2	11	2	3	3	3		
