# Supplementary material for: Genes Involved in Stress Response and Especially in Phytoalexin Biosynthesis Are Upregulated in Four Malus Genotypes in Response to Apple Replant Disease
Source: Front Plant Sci. 2020 Feb 28;10:1724. doi: 10.3389/fpls.2019.01724 (PMC7059805; doi:10.3389/fpls.2019.01724)
Supplement: Supplementary file 2 [file DataSheet_2.pdf]

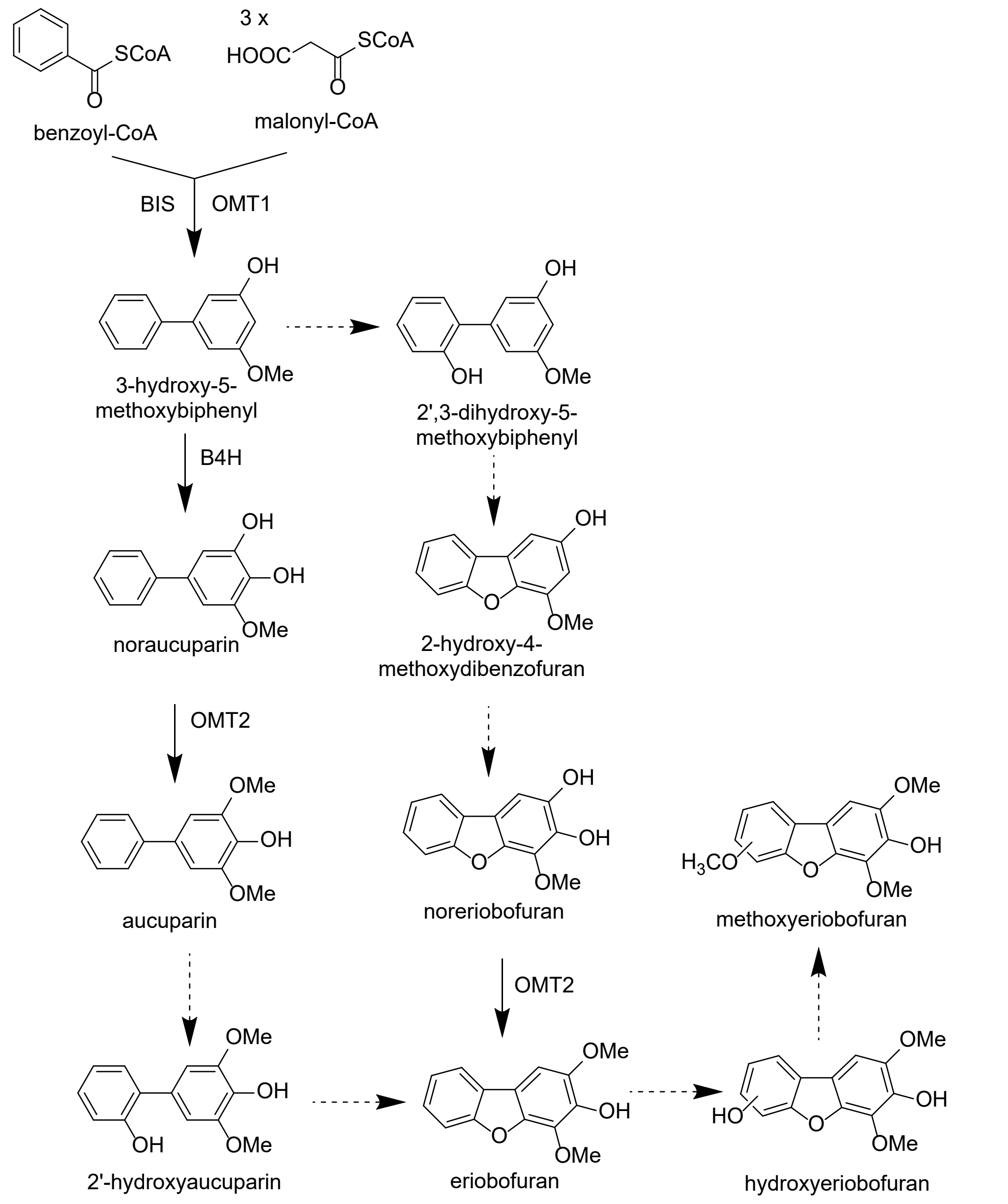

Figure S2: Proposed biosynthetic pathway of biphenyl and dibenzofuran phytoalexins. Solid arrows represent established steps whereas broken arrows mark hypothetical reactions. BIS, biphenyl synthase; OMT, *O*-methyltransferase; B4H, biphenyl 4-hydroxylase.
