# Supplementary material for: Genes Involved in Stress Response and Especially in Phytoalexin Biosynthesis Are Upregulated in Four Malus Genotypes in Response to Apple Replant Disease
Source: Front Plant Sci. 2020 Feb 28;10:1724. doi: 10.3389/fpls.2019.01724 (PMC7059805; doi:10.3389/fpls.2019.01724)
Supplement: Supplementary file 3 [file Table_1.docx]

**Table S1**: Experimental setup and derived replicates for gene expression and growth analysis

| **Number**  **of plants** | **Soil type and treatment** | | | | **Comments** |
| --- | --- | --- | --- | --- | --- |
|  | **Heidgraben** | | **Meckenheim** | |  |
|  | **ARD** | **γ ARD** | **ARD** | **γ ARD** |  |
| Total | 25 | 25 | 25 | 25 | - 100 plants of M26, B63, and MAL0595 each - 12 plants of M9 - 312 plants in total |
| Used after 7d | 15 | 15 | 15 | 15 | For gene expression analysis   - 5 samples (3 plants each) of roots and leaves = 40 samples of M26, B63 and MAL0595 = 120 in total - 2 samples only for Heidgraben soil (roots and leaves) of M9 = 8 - 128 samples in total |
| Used after 28 d | 9-10 | 9-10 | 9-10 | 9-10 | Growth parameter (9-10 plants)   - Only done for M26, B63 and MAL0595   Phytoalexin analysis (4-5 plants)   - Only done for M26, B63 and MAL0595 |
