# Supplementary material for: Genes Involved in Stress Response and Especially in Phytoalexin Biosynthesis Are Upregulated in Four Malus Genotypes in Response to Apple Replant Disease
Source: Front Plant Sci. 2020 Feb 28;10:1724. doi: 10.3389/fpls.2019.01724 (PMC7059805; doi:10.3389/fpls.2019.01724)
Supplement: Supplementary file 5 [file Table_3.docx]

**Table S3:** Plating of soil suspensions of ARD soil and γARD soil before and after storage for three months from the two sites (Heidgraben and Meckenheim). n = 6 Petri dishes from two independently plated samples. Colony-forming units (CUFs) were counted after one day for bacteria and seven days for fungi.

| **untreated** | **site** | **Bacteria (* 10^4^)** | | **Fungi (* 10^4^)** | |
| --- | --- | --- | --- | --- | --- |
|  |  | **before storage** | **after storage** | **before storage** | **after storage** |
|  | Heidgraben | 7.3 ± 2.1 | 6.3 ± 2.6 | 6.0 ± 3.7 | 2.2 ± 1.5 |
|  | Meckenheim | 52.5 ± 10.2 | 63.0 ± 43.9 | 1.7 ± 1.6 | 0.3 ± 0.5 |
| **γ-irradiated** |  | **Bacteria (* 10^2^)** | | **Fungi (* 10^2^)** | |
|  | **site** | **before storage** | **after storage** | **before storage** | **after storage** |
|  | Heidgraben | 0 | 0 | 0 | 0 |
|  | Meckenheim | 0 | 0 | 0 | 0 |
