# Supplementary material for: Genes Involved in Stress Response and Especially in Phytoalexin Biosynthesis Are Upregulated in Four Malus Genotypes in Response to Apple Replant Disease
Source: Front Plant Sci. 2020 Feb 28;10:1724. doi: 10.3389/fpls.2019.01724 (PMC7059805; doi:10.3389/fpls.2019.01724)
Supplement: Supplementary file 6 [file Table_4.docx]

**Table S4:** Dry masses of shoots and roots of the apple genotypes ‘M26, B63, and *Malus ×robusta* MAL0595 four weeks after transplanting to γARD soil and ARD soil from ‘Heidgraben’ and ‘Meckenheim’. Values with the same letter within one line did not differ significantly (Tukey test, *p* = 0.05, n = 4-5). StDV – standard deviation; Red - Growth reduction in % compared between gamma and untreated soil from one site; n.s. – not significant. Asterisks indicate a significant reduction regarding the Tukey test (* = *p* < 0.05).

| **Genotype** | **Dry mass ± StDV**  **[mg]** | **Heidgraben** | | | **Meckenheim** | | |
| --- | --- | --- | --- | --- | --- | --- | --- |
|  |  | **γARD** | **ARD** | **% Red** | **γARD** | **ARD** | **% Red** |
| **M26** | Shoot | 285.2 ± 69.5^a^ | 199.2 ± 57.2^a^ | -30.2 | 440.2 ± 129.9^b^ | 239.8 ± 63.3^a^ | -45.5^*^ |
|  | Root | 37.8 ± 7.8^n.s.^ | 32.0 ± 17.5^n.s.^ | -15.3 | 43.4 ± 10.2^n.s.^ | 24.4 ± 7.0^n.s.^ | -43.8 |
| **B63** | Shoot | 289.8 ± 71.7^ab^ | 174.8 ± 41.8^bc^ | -39.7 | 377.0 ± 90.9^a^ | 166.5 ± 12.0^c^ | -50.6^*^ |
|  | Root | 90.2 ± 36.2^a^ | 51.2 ± 14.1^ab^ | -43.2 | 48.0 ± 17.4^b^ | 34.5 ± 8.5^b^ | -28.1 |
| **MAL0595** | Shoot | 266.2 ± 63.2^n.s.^ | 197.5 ± 53.7^n.s.^ | -25.8 | 423.9 ± 284.4^n.s.^ | 254.2 ± 186.5^n.s.^ | -40.0 |
|  | Root | 77.4 ± 26.8^n.s.^ | 48.0 ± 17.8^n.s.^ | -38.0 | 70.5 ± 49.7^n.s.^ | 76.8 ± 62.6^n.s.^ | +8.9 |
